# Edema formation in congestive heart failure and the underlying mechanisms

**DOI:** 10.3389/fcvm.2022.933215

**Published:** 2022-09-27

**Authors:** Zaid Abassi, Emad E. Khoury, Tony Karram, Doron Aronson

**Affiliations:** ^1^Department of Physiology, Bruce Rappaport Faculty of Medicine, Technion–Israel Institute of Technology, Haifa, Israel; ^2^Department of Laboratory Medicine, Rambam Health Care Campus, Haifa, Israel; ^3^Department of Vascular Surgery and Kidney Transplantation, Rambam Health Care Campus, Haifa, Israel; ^4^Department of Cardiology, Rambam Health Care Campus, Haifa, Israel

**Keywords:** heart failure, edema, mechanisms, neurohumoral, cardiorenal syndrome, Na+ retention, renal venous congestion, intra-abdominal pressure

## Abstract

Congestive heart failure (HF) is a complex disease state characterized by impaired ventricular function and insufficient peripheral blood supply. The resultant reduced blood flow characterizing HF promotes activation of neurohormonal systems which leads to fluid retention, often exhibited as pulmonary congestion, peripheral edema, dyspnea, and fatigue. Despite intensive research, the exact mechanisms underlying edema formation in HF are poorly characterized. However, the unique relationship between the heart and the kidneys plays a central role in this phenomenon. Specifically, the interplay between the heart and the kidneys in HF involves multiple interdependent mechanisms, including hemodynamic alterations resulting in insufficient peripheral and renal perfusion which can lead to renal tubule hypoxia. Furthermore, HF is characterized by activation of neurohormonal factors including renin-angiotensin-aldosterone system (RAAS), sympathetic nervous system (SNS), endothelin-1 (ET-1), and anti-diuretic hormone (ADH) due to reduced cardiac output (CO) and renal perfusion. Persistent activation of these systems results in deleterious effects on both the kidneys and the heart, including sodium and water retention, vasoconstriction, increased central venous pressure (CVP), which is associated with renal venous hypertension/congestion along with increased intra-abdominal pressure (IAP). The latter was shown to reduce renal blood flow (RBF), leading to a decline in the glomerular filtration rate (GFR). Besides the activation of the above-mentioned vasoconstrictor/anti-natriuretic neurohormonal systems, HF is associated with exceptionally elevated levels of atrial natriuretic peptide (ANP) and brain natriuretic peptide (BNP). However, the supremacy of the deleterious neurohormonal systems over the beneficial natriuretic peptides (NP) in HF is evident by persistent sodium and water retention and cardiac remodeling. Many mechanisms have been suggested to explain this phenomenon which seems to be multifactorial and play a major role in the development of renal hyporesponsiveness to NPs and cardiac remodeling. This review focuses on the mechanisms underlying the development of edema in HF with reduced ejection fraction and refers to the therapeutic maneuvers applied today to overcome abnormal salt/water balance characterizing HF.

## Introduction

Generalized edema, the main clinical characteristic of extra cellular fluid (ECF) volume expansion, represents pathological sodium balance, and constant accumulation of water in excessive volumes in the interstitial compartment (1). It occurs in various edematous disease states including congestive heart failure (CHF), cirrhosis with ascites, and nephrotic syndrome. Regardless of its etiology, heart failure (HF) is primarily classified into two subgroups according to the ventricular ejection fraction (EF), namely HF with reduced EF (HFrEF) and HF with preserved EF (HFpEF). The prevalence of both HFrEF and HFpEF is roughly equal among HF patients, and both have a similar ominous prognosis, yet each subgroup exhibits unique clinical features and different responses to medical intervention (2, 3). HF is a clinical setting characterized by the incapability of the heart to perfuse enough blood and oxygen/nutrition to peripheral tissues. This happens frequently in low-output CHF. In response to these alterations, a series of compensatory circulatory and neurohormonal adjustments take place in order to maintain blood pressure and perfusion to various vital organs including the brain, lungs, and kidneys (4). In the early stages, these adaptations are beneficial and fulfill their compensatory role (compensated CHF). However, as CHF evolves, exaggerated stimulation of these systems becomes harmful as evident by profound systemic vasoconstriction and increased loading in the failing heart, eventually leading to the development of decompensated CHF (4, 5). Among the major abnormalities at the later stage is the disorder in the effector arm of volume control, where disproportionate activation of vasoconstrictor-sodium retaining systems, along with the failure of vasodilatory natriuretic factors, take place resulting in excessive salt and water balance (4, 5). Early and even before clinical cardiac failure manifestations develop, underlying renal aberrations limit their natriuretic response (6–9). This behavior agrees with the concept that the primary disturbance underlying sodium retention does not originate within the kidneys. Rather, renal sodium retention is secondary to circulatory disturbance provoked by the failing heart (10–12). The evolvement of the latter activates vasoconstrictive and anti-natriuretic systems that continue to retain sodium/water despite the subtle or overt expansion of ECF volume (13, 14).

The purpose of this review is to summarize the current understanding of the disturbances in the mechanisms that occur in one of the most widespread edema-forming states, namely CHF, and the derived therapeutic options applied today to overcome the elevated salt balance and edema characterizing this clinical setting.

## Mechanisms underlying edema formation

### Alterations in starling forces and interstitial fluid accumulation

Trans-capillary convective fluid flow and diffusive solute transport occur in CHF (1, 4, 15). The water movement is derived from hydrostatic and osmotic pressure gradients (5). Capillary hydraulic pressure is determined by several factors, such as arterial and venous blood pressures (SBP and CVP, respectively), blood flow, and resistances enacted by the pre- and post-capillary sphincters. While SBP is influenced by cardiac output (CO), systemic vascular resistance, and intra-vascular filling, systemic venous pressure is controlled by right atrial pressure, intra-vascular volume, and venous capacity. The latter hemodynamic parameters are profoundly dependent on sodium balance. Specifically, expansion of interstitial compartment can directly attenuate venous compliance and hence alter overall cardiovascular performance (16). Normally, the interstitial fluid pressure is sub-atmospheric, therefore, even a small increase in the volume of this ECF sub-space tends to enhance tissue hydraulic pressure, which opposes the movement of fluid into the interstitial compartment (17). Collectively, the development of generalized edema may stem from disorders in microcirculatory hemodynamics, where elevated venous pressure transmitted to the capillary is of major relevance to CHF as substantial renal fluid and sodium retention and increased ECF volume are hallmark features of this disease.

### Perturbations of the afferent limb of volume homeostasis in congestive heart failure

The fact that the kidneys’ ultrastructure is normal in CHF and keeps retaining sodium and water avidly, despite ECF expansion, may stem from either “backward failure” or “forward failure”. The former indicates that the volume sensing mechanisms fail to appropriately detect the elevated circulating volume. According to this theory, the failing heart results in venous congestion along with increased capillary pressure, where both provoke fluid accumulation in the interstitium concomitantly to plasma volume depletion. Attenuation of plasma volume stimulates renal sodium and water retention. The concept of “forward failure” underscores the contribution of the myocardium failure in supplying sufficient blood to the various tissues including the kidneys, which are no longer able to maintain normal sodium excretion. Noteworthy, both theories emphasize the “underfilling of the arterial circulation”, reduced cardiac output, and activation of neurohormonal responses (18–24). Therefore, a unifying hypothesis termed “arterial underfilling” was established to explain the sustained sodium and water retention by the kidneys in response to diminished cardiac output (10–12). In this context, hemodynamic alterations, and activation of neurohormonal compensatory systems in CHF, are similar to those seen in true dehydration. However, it should be emphasized that in contrast to real hypovolemia, CHF is characterized by elevated intracardiac pressures, which are supposed to stimulate the release of natriuretic peptides (NPs), namely atrial NP (ANP) and brain NP (BNP), and eventually provoke natriuretic and diuretic responses. The blunted natriuresis characterizing CHF may stem from interrupted signaling in afferent sensing sites localized to the cardiopulmonary system. These include disrupted baroreceptors located in the carotid sinus and aortic arch, besides malfunctioning of mechanosensitive nerve endings localized in cardiac chambers and cardiopulmonary system. Both arterial baro- and cardiopulmonary reflexes are blunted in CHF, as expressed by an inadequate tonic inhibitory effect on sympathetic outflow and eventually sympathetic nervous system (SNS) activation, together with anti-diuretic hormone (ADH) and renin secretion along promoting renal retention of salt and water despite of volume expansion (25, 26). In this context, it was shown that the interaction between volume sensing and urinary sodium excretion is maintained in compensated CHF (27), but not decompensated CHF, as was previously shown by Abassi et al. in an experimental model of heart failure induced by arteriovenous (A-V) fistula (13). Furthermore, important defects in the interaction between the cardiac and carotid baroreceptors and renal sympathetic activity have been reported in CHF. In this context, DiBona et al. (28) demonstrated a higher renal efferent sympathetic activity in rats with experimental CHF induced by left anterior descending (LAD) artery ligation. Interestingly, the renal sympathetic nerve hyperactivity of these animals was not attenuated following volume expansion. Moreover, the same group demonstrated that the aberrant regulation of renal sympathetic activity was associated with the disrupted function of cardiac, pulmonary, and arterial baroreceptors (29).

### Abnormalities in efferent limb of volume homeostasis in congestive heart failure

Besides the aberrant sensing mechanisms, CHF is associated with the activation of several adaptive alterations in volume control. As mentioned above, these effector mechanisms include activation of neural, humoral, and paracrine systems that impose changes in glomerular hemodynamics and tubular transport, which in turn lead to avid sodium retention (1). On the other hand, CHF is also characterized by the activation of vasodilatory natriuretic systems, aimed at opposing the vasoconstrictor anti-natriuretic factors. Thus, the net effect on sodium and water balance in CHF is determined by the balance between these antagonistic systems. Although activation of these vasodilatory and natriuretic systems is essential to counterbalance the vasoconstrictor/antinatriuretic systems, abnormal and continuous activation of the efferent limb of volume control along blunted renal action of NPs profoundly contribute to the classic manifestations of CHF and further deterioration of cardiac function (1).

### Alterations in glomerular hemodynamics

**FIGURE 1:**
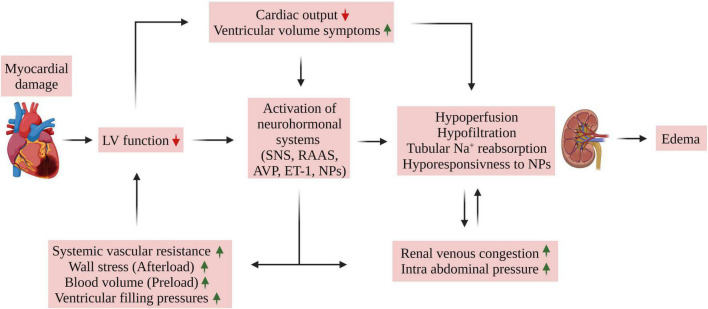
Mechanisms of edema formation in HFrEF. Myocardial damage of various etiologies may lead to cardiac dysfunction as evident by reduced cardiac output and ejection fraction. The resultant reduced organ blood flow promotes activation of neurohormonal systems (SNS, RAAS, AVP, and ET-1) which leads to salt and fluid retention, often exhibited as pulmonary congestion, peripheral edema, dyspnea, and fatigue. Unique relationship between the heart and the kidney plays a central role in this phenomenon. Specifically, the interaction between the heart and kidney in HF is complex and involves multiple interdependent mechanisms which includes (1) hemodynamic alterations resulting in insufficient peripheral and kidney perfusion, (2) HF is characterized by elevated central venous pressure (CVP), which is associated with renal venous hypertension/congestion along with increased intra-abdominal pressure (IAP). The latter was shown to reduce RBF, leading to a decline in GFR. Moreover, persistent activation of neurohormonal factor along with reduced renal response to NPs aggravates the hypoperfusion and hypofiltration due to their vasoconstrictive and tubular Na+ and H_2_O retaining properties which further aggravates CVP and IAP and eventually the development of edema. ANP, atrial natriuretic peptide; BNP, brain natriuretic peptide; AVP, arginine vasopressin; ET-1, endothelin 1; LV, left ventricle; NPs, natriuretic peptides; RAAS, renin angiotensin aldosterone system; SNS, sympathetic nervous system.

The interplay between the heart and kidneys in CHF is complex (Figure 1), involving multiple interdependent mechanisms, which can be divided into four categories (6–9): (1) Insufficient peripheral blood flow during HF results in deleterious alterations in renal hemodynamics as evident by increased renal vascular resistance, reduced glomerular filtration rate (GFR), and a marked reduction in renal plasma flow (RPF) resulting in increased filtration fraction (FF). This phenomenon was observed in rats with CHF induced by left coronary ligation (30), where CHF rats exhibited lower single nephron GFR (SNGFR) than control animals. Micropuncture assessment revealed a reduction in single nephron plasma flow (SNPF) which was to a greater extent than the decline in SNGFR, accounting for a higher single nephron filtration fraction (SNFF). The preferential decline in SNPF as compared with SNGFR is attributed to vasoconstriction of both afferent, and especially efferent arterioles. Similar alterations in glomerular hemodynamics have been also reported in rats with A-V fistula, a high output failure model (31). As a result, colloid osmotic pressure gradient (Δπ) enhances over the glomerular capillary which eventually leads to impaired GFR as the hydrostatic pressure gradient (ΔP) is decreased too. Since the kidneys receive about one-quarter of CO and considering that GFR is dependent on RPF, renal hypoperfusion can lead to renal hypoxia; (2) HF is characterized by elevated CVP, which is associated with renal venous hypertension (32). The latter was shown to reduce RBF in animal models, leading to a decline in GFR (4); (3) Activation of neurohormonal factors, where the reduced CO and the subsequent decline in blood pressure and renal perfusion activate the renin-angiotensin-aldosterone-system (RAAS) and SNS. Activation of these neurohormonal systems results in deleterious effects on both the kidneys and the heart, including sodium and water retention, systemic and renal vasoconstriction, elevated venous volume/return, and enhanced oxidative stress. Moreover, angiotensin II (Ang II) and aldosterone promote cardiac and renal remodeling (33, 34). In addition, HF is characterized by increased production of endothelin-1 (ET-1) and ADH following baroreceptor activation (7), both of which enhance systemic vasoconstriction and reduce free water clearance; (4) secretion of various factors that play an important role in the deterioration of the cardiac and renal function along with systemic and local inflammation, endothelial dysfunction, anemia, and other metabolic alterations (6, 35). In summary, worsening renal function (WRF) during acute decompensated HF occurs mainly due to systemic hemodynamic derangements, such as increased venous pressure, elevated intra-abdominal pressure (IAP), and drop in arterial blood pressure along with diminished cardiac function (see the following sections).

### Tubular sodium retention

**FIGURE 2:**
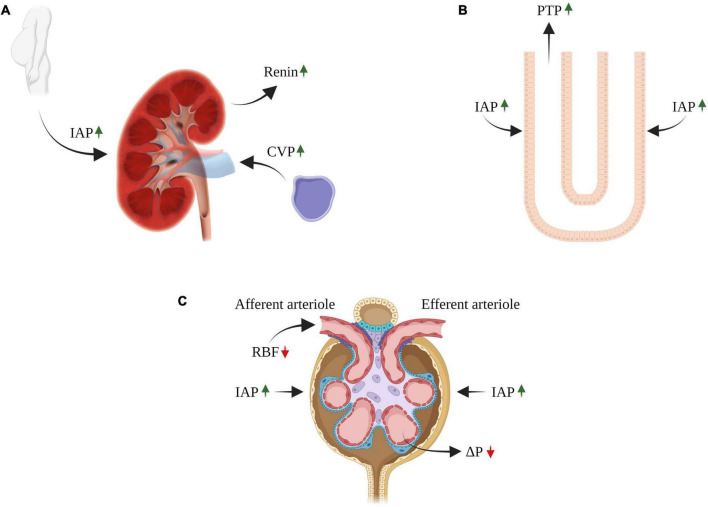
Impact of congestive venous pressure (CVP) and increased intra-abdominal pressure (IAP) on kidney function in heart failure. Elevated CVP is transmitted back to the renal veins leading to renal dysfunction **(A)**. The contribution of renal venous congestion to renal dysfunction in HF is complex and involves multiple mechanisms, including increased pressure along the renal vasculature without decline in ΔP, decreasing the net pressure gradient filtration pressure (NFP) across the glomerulus and thereby reduced GFR **(C)**. In addition, the increase in renal venous pressure can increase intrarenal interstitial pressure leading to compression of the tubules, increased tubular fluid pressure **(B)**, with reduced GFR due to an increase in hydrostatic pressure in the Bowman’s capsule **(C)**. In addition, reduced GFR and sodium excretion may develop secondary to intra-abdominal hypertension (IAH), a hallmark feature of decompensated CHF **(A–C)**. The diverse deleterious renal effects of elevated IAP may overlap with those of venous congestion. There is a direct compression of abdominal contents that result in a prominent reduction in RBF (compression of renal arteries) and elevation in renal parenchymal and renal vein pressures **(A–C)**. RBF, renal blood flow; GFR, glomerular filtration rate; CVP, central venous pressure; IAP, intraabdominal pressure; PTP, proximal tubular pressure. ΔP, hydrostatic pressure gradient.

The adverse alterations in glomerular hemodynamics, WRF, impaired tubular flow, and hormonal status characterizing decompensated CHF enhance tubular reabsorption of sodium at both the proximal nephron and collecting duct (4). As mentioned above, the elevated Δπ along the decreased ΔP through the glomerular capillary length favors fluid movement from the tubular lumen into the capillary, and sodium and water reabsorption in the proximal tubule to peritubular capillaries. Since the kidneys are encapsulated, renal venous congestion elevates the interstitial hydrostatic pressure in both the kidneys, peritubular capillaries, and tubuli (Figure 2) (36). Likewise, the enhanced renal lymphatic flow characterizing CHF reduces interstitial π, thus aggravating tubular salt reabsorption (37). The latter is manifested by avid proximal sodium reabsorption as a result of abnormal glomerular hemodynamics and activation of neurohormonal systems as have been shown in both experimental and clinical studies (4, 38). Evidence for exaggerated proximal sodium reabsorption conjugated with low delivery of sodium to more distal tubuli during CHF induced by LAD ligation was derived from clearance experiments where mannitol was infused (30, 39), inhibition of distal sodium reabsorption (40) and deoxycorticosterone acetate (DOCA) escape (41). Further support for this notion came from the findings that restoring the increased SNFF with angiotensin-converting enzyme (ACE) inhibitor normalized proximal peritubular capillary starling forces and sodium reabsorption (41). Yet, direct actions of Ang II and norepinephrine (NE) secreted from the renal nerve contribute to the enhanced proximal sodium reabsorption (4). In this regard, both Ang II and NE may act by modulating both renal hemodynamics, as well as by directly boosting proximal sodium epithelial transport, thus augmenting the overall proximal sodium reabsorption capacity (4). Besides the proximal tubule, the distal nephron site also takes part in the exaggerated tubular sodium reabsorption in experimental models of CHF. Specifically, applying micropuncture technique in experimental high or low CO CHF revealed enhanced distal nephron sodium reabsorption (42–45). Moreover, dogs with CHF induced by vena cava constriction cannot excrete sodium load due to exaggerated sodium reabsorption by loop of Henle (46). In this context, reduced medullary blood flow due to vasoconstriction prevents washout of solutes from the renal medulla, thus leading to reduced free water excretion and consequently to impaired urinary dilution (47).

### Humoral mechanisms

Most hospitalized CHF patients display a variable degree of volume overload (48, 49). This is largely attributed to the mobilization of compensatory anti-natriuretic and vasoconstrictive systems including RAAS, SNS, ADH, and endothelins (ETs), which enhance vascular resistance and promote salt and water reabsorption by the kidneys (4). These deleterious actions overcome the activated vasodilatory/natriuretic substances, such as NPs, nitric oxide (NO), prostaglandins (PGs), adrenomedullin (AM), and urotensin II (UII) (13). It is well accepted that salt and water balance is largely determined by a fine balance between these antagonistic systems, namely vasoconstrictive/anti-natriuretic and vasodilator/natriuretic substances. The development of excessive sodium balance and generalized edema in CHF represents a milestone where the balance is in favor of the vasoconstrictive/anti-natriuretic systems (Figure 1). However, up to 60% of HF patients suffer from a certain degree of kidney dysfunction (GFR < 60 ml/min), which reduces their ability to excrete excessive amounts of sodium (32). This subgroup of CHF patients became of special interest in the last few years, as numerous studies uncovered that kidney dysfunction in CHF is a stronger predictor of mortality than impaired cardiac performance (50–54). Collectively, these findings confirm that WRF is common in HF and has been associated with decreased survival, a high rate of hospitalization, and disease progression (53, 54).

### Neurohormonal systems

#### Vasoconstrictive/anti-natriuretic systems

##### Renin-angiotensin-aldosterone system

The renin-angiotensin-aldosterone system (RAAS) plays a critical role in the homeostasis of ECF volume, sodium balance, blood pressure, and cardiac performance (55, 56). This sodium/water-retaining and vasoconstrictive system is activated in hypovolemic and hypotensive conditions which compromise hemodynamic stability, such as hemorrhage, hypotension, dehydration, low sodium intake, and activation of SNS. Our understanding of the RAAS has been evolving over the last 120 years (57, 58). Classically, the RAAS is considered as a paracrine/endocrine axis involved in tubular sodium reabsorption and vasoconstriction, thus may eventually lead to the development of hypertension and target organ damage with concomitant inflammation, oxidative stress, cell proliferation, apoptosis, fibrosis, and coagulation (57, 59). The system cascade is initiated and regulated by the classic renin activity originating from the kidneys (Granular cells) (60), but also expressed locally in the heart during HF (61–64). Renin produces angiotensin (Ang) I, an inactive 10 amino acid (aa) peptide from circulating angiotensinogen. Ang I is then converted by ACE to Ang II, an 8 aa active peptide. ACE is primarily localized to the endothelial cells of the pulmonary vasculature. The deleterious impact of RAAS is attributed to its main component, namely Ang II, where it exerts its adverse actions by binding to angiotensin II receptor type 1 (AT1R) expressed in various target organs including the kidneys, heart, and blood vessels. The development of ACE inhibitors (ACEi), and later of AT1R blockers (ARBs), revolutionized the therapeutic approach to kidney and heart diseases (65–68), where both groups of RAAS blockers attenuate cardiac and renal remodeling and slow down the progression of HF (69–72) and chronic kidney disease (CKD) (73–75). Aldosterone, the other active component in the RAAS cascade, was also found to enhance sodium retention by the principle cells localized to the collecting duct, thus contributing to ECF expansion and independently accelerating organ fibrosis, a process revised by aldosterone antagonists (76).

However, the complexity of the RAAS has been unraveled in the last two decades, where numerous studies reported that besides the adverse ACE/Ang II/AT1R axis (the “pressor arm”), there is a beneficial and protective pathway that attenuates the adverse vasoconstrictory and salt-retaining effects of the abovementioned axis (the “depressor arm”) (77, 78). Specifically, our attention was shifted to another component of the RAAS, namely angiotensin-converting enzyme 2 (ACE2). The latter converts Ang II to the bioactive 7-amino-acid peptide, Ang 1–7. Moreover, ACE2 converts Ang I into Ang 1–9, which can be further converted to Ang 1–7 by ACE. Additional pathway of Ang 1–7 production involves neural endopeptidase, or neprilysin (NEP), which converts Ang I directly into Ang 1–7 (79–84).

Ang II exerts its anti-diuretic and anti-natriuretic effects through the regulation of both renal hemodynamic and tubular sodium and water transport (55). Specifically, Ang II pressor action on the kidney decreases renal blood flow and urinary sodium excretion. In this context, Ang II binds to AT-1 receptors localized to renal vascular smooth muscle cells, causing vasoconstriction of both the afferent and efferent arterioles, resulting in kidney hypoperfusion. Moreover, activation of AT-1 receptors in the brain increases cardiac and vasculature sympathetic output, thus increasing CO and total peripheral resistance along elevated blood pressure. In addition, Ang II provokes ADH release from the posterior pituitary gland, which in turn enhances water retention by the collecting duct to maintain blood volume. Finally, Ang II stimulates thirst, where the increased water intake increases ECF including blood volume, which collectively raises blood pressure (55). The elevated circulatory levels of Ang II or local within-organ RAAS activation in HF (85) aggravates tubular handling of salt and water, by directly stimulating proximal tubular sodium absorption and indirectly *via* increasing the release of aldosterone and endothelin, and stimulating thirst despite a typically low serum osmolality (86). Therefore, blockade of the RAAS in HF patients with reduced ejection fraction (EF) by administration of either ACE inhibitors or ARBs can facilitate sodium excretion although can deteriorate renal hemodynamics and eventually kidney function due to efferent arteriole vasodilation (54, 87, 88). In this regard, a meta-analysis of published study data by Beldhuis (89) on 28,961 patients revealed that RAAS inhibitors induce WRF in both HFrEF and HFpEF. The latter group exhibited an increased mortality risk when placed on RAAS inhibitors. In contrast, WRF in HFrEF following treatment with ACE inhibitors increased the mortality rate to a lesser extent as compared with HFpEF patients on RAAS inhibitor-induced WRF.

ACE2 is a transcellular protein, which is abundantly expressed in several vital organs, such as the intestine, kidney, endothelial cells, heart, lung, brain, and testis (80). In the heart, ACE2 is widely expressed in the endothelium, smooth muscle cells, and cardiac myocytes (90). As mentioned above, locally expressed ACE2 converts Ang II to Ang 1–7 (81), which exerts vasodilatory, natriuretic/diuretic, anti-inflammatory, and anti-fibrotic effects *via* Mas receptor (MasR) (84). Notably, both clinical and experimental HF displays upregulation of cardiac ACE2 and enhanced Ang 1–7 production, which may represent a cardioprotective compensatory mechanism aimed at counterbalancing the adverse effects of ACE/Ang II/AT1R axis on the myocardium (84, 90, 91). In this context, Tripathi et al. have demonstrated that ACE2/Ang 1–7/MasR exerts a protective role toward systemic and pleural edema suppression along with enhanced survival in mice model of HFrEF with progressive dilated cardiomyopathy (DCM), without comorbidities from modulated blood pressure and renal failure (92).

In summary, Ang II, the main effector of the RAAS, controls ECF and urinary sodium excretion *via* renal hemodynamic and tubular actions, along with systemic vasoconstriction, as well as aldosterone and ADH release mechanisms. The intra- and extra-renal actions of Ang II are exaggerated under a variety of disease states, such as HF, thus aggravating cardiac dysfunction and myocardial remodeling. The presence of ACE2/Ang 1–7/MasR may act as a protective arm to face the deleterious arm of the RAAS, namely, ACE/Ang II/AT1-R axis.

##### Sympathetic nervous system

Renal sympathetic nerves innervate the vasculature and nephrons, where they play a central role in the regulation of renal hemodynamics and tubular function (93, 94). Specifically, sympathetic nerve endings were observed in smooth muscle cells of renal vessels, mesangial cells, juxtaglomerular granular cells, and the various tubular segments, including proximal convoluted, Henle’s loop, and distal tubuli. The early stages of HF are characterized by high sympathetic activity and high levels of circulatory NE, which harmfully affect vital organs, including the heart and kidneys (95). As mentioned above, the activation of neurohormonal systems during HF is initially beneficial in maintaining systemic blood pressure (BP) and adequate organ perfusion, however, it becomes detrimental as the disease evolves (96). Increased renal sympathetic nerve outflow attenuates urinary sodium and water excretion through (1) exaggerated tubular sodium reabsorption throughout the nephron; (2) hypoperfusion and hypofiltration by inducing afferent and efferent arteriole vasoconstriction; and (3) provoking renin secretion from the juxtaglomerular granular cells (Figure 2), which eventually leads to RAAS activation (60) and attenuation of the renal actions of ANP (97). Support for the major contribution of the renal nerve to renal excretory and hemodynamic derangement was derived from experimental HF studies showing decreased sodium retention, hyperperfusion, hyperfiltration, and vasodilation of both afferent and efferent arterioles following renal denervation (95, 97, 98). In line with these findings, α or β receptors antagonists also enhanced urinary excretion of sodium and water, probably secondary to improvement in both renal and cardiac hemodynamics and suppression of renal sympathetic nerve, as well as RAAS activity (1, 99), although this matter remains somewhat controversial (100). Collectively, activation of the renal sympathetic nerve composes a major factor in the anti-natriuretic and vasoconstrictive systems which largely contributes to avid renal sodium and water retention characterizing patients with advanced HF (101).

##### Anti-diuretic hormone

Since the pioneer report by Szatalovicz et al. in the early 80s (102), several studies have reported elevated circulatory levels of ADH in patients with HF, especially in those with advanced CHF and hyponatremia (1). The increased secretion of ADH in these patients is attributed to non-osmotic stimuli such as attenuated compliance of the left atrium and activation of the baroreceptors and RAAS (101, 103). This non-osmotic release of ADH is largely responsible for hyponatremia, a clinically important complication of heart failure, as ADH stimulates V2 receptors (V2) along increased aquaporin-2 AQP-2 water channel density in the apical side of epithelial cells in the collecting duct, ensuing water retention and eventually hyponatremia. The contribution of ADH to the excessive water balance in CHF is supported by the findings that oral treatment with V2 antagonists (Aquaretics) provoked significant free water clearance, low urinary osmolarity, and elevation of plasma osmolality, along with downregulation of AQP-2 in the collecting duct (104). Besides the activation of the V2/AQP-2, ADH also stimulates V1a receptors localized to the vascular smooth muscle cells, with constriction of coronary vessels and stimulation of cardiac myocyte proliferation (101, 103). These findings suggest an adverse role for vasopressin in fluid overload and cardiac remodeling characterizing CHF patients. This notion is supported by clinical trials demonstrating that V2 blockade induces diuresis and lowers congestion without WRF when administered with furosemide to chronic HFrEF, but unfortunately did not improve outcomes when applied during the post-acute phase (105). In summary, these results implicate ADH in the pathogenesis of water retention and hyponatremia characterizing CHF, and that V2 receptor blockade may bear potential therapeutic properties for clinical CHF.

##### Endothelin

The endothelin family contains three members, namely endothelin-1 (ET-1), the most famous representative, endothelin-2 (ET-2), and endothelin-3 (ET-3). These peptides are generated and secreted mainly by endothelial cells, and act in the proximity of their production in a paracrine/autocrine mode of action *via* endothelin receptors A and B (ETA and ETB, respectively). Besides their explicit role in normal physiology, ETs are involved in the pathogenesis of many diseases, including cardiovascular and renal disorders (106, 107). Under normal conditions, ET-1 regulates basal vascular tone, glomerular hemodynamics, and sodium homeostasis. At the cardiac level, ET-1 synthesis takes place in cardiac myocytes, where it exerts positive inotropic effects at low doses, but can cause a reduction in CO at high concentrations. In addition, numerous studies have demonstrated that the kidney is a major site of ET-1 synthesis (mainly the inner medulla), besides being a preferential target organ of this peptide. Specifically, ET-1 exerts various effects on the kidneys, where it affects renal function by modulating: (1) renal vascular resistance; (2) tubular salt and water reabsorption; and (3) tonus, proliferation, and mitogenesis of mesangial cells (106). Noteworthy, this system is also involved in various pathophysiological conditions including hypertension, myocardial hypertrophy, and inflammation, and in the development and progression of renal and cardiovascular diseases, including CKD and CHF (108). Concerning the latter, ET-1 plays a role in cardiac remodeling *via* increasing fibroblast activation and inflammation in the failing heart or secondary to RAAS activation (108). Furthermore, the endothelin system is involved in kidney dysfunction in both acute and chronic kidney failure by inducing deleterious actions such as oxidative stress, inflammation, renal remodeling, interstitial fibrosis, glomerulosclerosis, reduced RBF and GFR, and water and sodium retention (109, 110). It should be emphasized that CKD and persistent congestion influence HF prognosis as was demonstrated in patients hospitalized with acute HF (111). Interestingly, the prognostic impact of these two parameters is associated with increased cytokine levels, suggesting an adverse role of inflammation in the prognostic impact of congestion and CKD and may also interfere with the outcome of these patients (111).

The pathophysiological involvement of ET-1 in CHF is supported by a few observations: (1) several studies have documented upregulation of the ET system in CHF (112). (2) Both experimental and clinical studies have reported that ET-1 receptor antagonists improved the severity of this disease state. Considering that CHF is associated with reduced renal perfusion along increased vascular resistance and elevated levels of ET-1, it is tempting to suggest a cause-and-effect relationship between the adverse alteration in renal hemodynamics and the activation of ET-1 in this clinical setting. Indeed, experimental studies have shown that administration of bosentan, a mixed ETA/ETB receptor antagonist, into rats with severe decompensated CHF induced by placement of aortocaval fistula remarkably improved RBF, as was evident by enhancement in renal cortical perfusion (113). In line with these findings, applying tezosentan, a dual ETA/ETB antagonist, in rats with CHF induced by myocardial infarction abolished the enhanced renal vascular resistance (RVR) and improved RBF and urinary salt excretion (114). These encouraging observations were backed up by several studies that have shown beneficial effects of chronic selective ETA blockers (115, 116) or dual ETA/ETB receptor antagonists (117) in experimental CHF, as was evident by relieving sodium retention and mitigating renal hypo filtration. Unfortunately, comprehensive clinical trials failed to show beneficial effects on morbidity and mortality (108). What else, fluid retention and elevated serum transaminase levels were important adverse effects of ET receptor antagonist agents, especially when using non-selective compounds (118). Therefore, proving the involvement of the ET system in the deranged renal hemodynamic and impaired excretory function in CHF and its therapeutic relevance in this clinical setting requires further study.

#### Vasodilatory and natriuretic systems

##### Natriuretic peptides

The natriuretic peptides (NPs) system plays a crucial role in maintaining cardio-renal homeostasis by opposing the abovementioned vasoconstrictor, anti-diuretic, anti-natriuretic, and tissue remodeling factors/pathways (119–121). The NP system includes two cardiac hormones, ANP and BNP. Under normal conditions, ANP and BNP are expressed mainly in the heart, with the appendages of the atria being the major site. Previous studies have shown that ANP and BNP encoding genes (*NPPA* and *NPPB*, respectively) play an important role already in the evolvement of the murine fetal heart (122). *NPPA* and *NPPB* lead to the translation of preprohormones (preproANP and preproBNP, respectively). Cleavage of the signaling tail of these peptides produces the inactive prohormones that are designated to undergo further cleavage by corin and furin enzymes to produce the potent peptides ANP and BNP, consisting of 28 and 32 amino acids, respectively (123–126). Both ANP and BNP contain an N-terminal tail and a 17 amino acid ring linked by a disulfide bond. The latter is crucial for the peptides’ biological activity. An additional C-terminal extension grants the peptides the ability to signal through the NP receptor. In the atria, the mature and activated BNP, and the proteolytic product N-terminal proBNP (NT-proBNP), together with the unprocessed proANP, are stored intracellularly within mature vesicles, which serve as a warehouse for regulated basal secretion of NPs (119). However, stimulated secretion of ANP and BNP occurs *via* three pathways. Atrial/ventricular wall distention and intracardiac volume overload enhance NPs secretion through the G_*o/i*_α-coupled receptor, resulting in higher levels of ANP and BNP in plasma (127). Through the G_q_α-coupled receptor, several secretagogues, like Ang II, phenylephrine, and ET-1, may induce the same effect (128). In contrast, inflammatory and bacterial lipopolysaccharides may augment BNP, but not ANP secretion, through different pathways leading to p38 activation (129). Interestingly, studies have demonstrated that stimulated secretion of ANP and BNP happens in a constitutive-like manner, meaning *de novo* ANP and BNP are secreted first, and preferentially *via* immature, rather than mature vesicles, as opposed to basal secretion (130).

A third hormone in the NPs system is named C-type NP (CNP). CNP is expressed mainly in the central nervous system, vascular endothelial cells, and kidney, where it functions primarily as a local autocrine/paracrine hormone. Despite its structural similarity with ANP and BNP, CNP lacks a C-terminal extension, and thus does not have natriuretic activity, and its secretion is not regulated by the heart.

The NPs exert their biological activities through natriuretic peptide receptors NPR-A and NPR-B. ANP and BNP recognize and bind NPR-A, and in a considerably less affinity to NPR-B. The latter constitutes the major binding receptor for CNP. NPR is a transmembrane receptor containing an intracellular domain with guanylyl cyclase (GC) activity. After binding to the receptor, ANP/BNP induces a conformational change in NPR-A leading to the activation of GC and a subsequent elevation in intracellular, plasma, and urine cGMP levels (119). By activating the GC in the various target tissues, ANP and BNP induce numerous effects, including natriuresis, diuresis, anti-fibrosis, anti-proliferation, and vasorelaxation, in addition to lowering blood pressure and cardiac preload. A third receptor, NPR-C, lacks the GC-coupled intracellular domain and acts as a clearance receptor through the internalization and degradation of NPs (119).

###### Natriuretic peptides blunted response in heart failure

In HF, ANP, and BNP secretion is significantly enhanced, and their plasma levels are substantially elevated (131–133). Today, NT-proBNP and BNP plasma levels serve as clinical biomarkers of choice for diagnosing acute decompensated heart failure (ADHF) and are also used as prognostic biomarkers (33, 134–136). An international study suggested incorporating NT-proBNP as a continuous measure along with other clinical variables to provide a more consistent, accurate, and individualized approach to HF patients (137). Nonetheless, one of the paradoxes seen in HF patients is the attenuated NPs effects despite their exceptionally elevated circulating levels in the plasma (138, 139). This phenomenon of NPs resistance/blunted response in HF has been of great interest for researchers in the last two decades, and many mechanisms have been suggested to explain the apparent paradox as outlined below:

*Hemodynamic alterations*: Hemodynamic changes and the decrease in CO along with a subsequent decline in renal perfusion occurring in HF, which have been widely studied and are well defined (6, 140), might be of paramount significance in the pathophysiology of renal hypo-responsiveness to NPs. NPs have been shown to undergo free filtration in the glomeruli and exert their biological effects in renal tubuli. In addition, HF syndrome is characterized by substantially elevated neurohormonal factors including Ang II, aldosterone, ADH, ET-1, and the SNS, all of which contribute to the attenuated renal and systemic effects of ANP and BNP (see above). Thus, poor renal blood perfusion, and the state of neurohormonal overactivation, may be key players in the attenuated effects of NPs in the kidney.

*Post-translational modifications of natriuretic peptides*: The abundance of the less-active forms of natriuretic peptides can be a result of post-translational modifications, which have been demonstrated by several studies to have the ability to modulate the activity, stability, and potency of NPs. This important fine-tuning ability can be achieved by the process of O-glycosylation of specific amino acids within the peptide (141–144). Semenov et al. (123, 145) have shown that the processing of proBNP by HEK293 cells expressing human furin and corin enzymes is suppressed by the *O*-glycosylation at threonine 71 amino acid located closely to the cleavage site. Furthermore, when incubated with purified furin, *O*-glycosylated proBNP extracted from the plasma of HF patients was significantly less activated when compared to non-glycosylated proBNP.

In another study, *O*-glycosylation modifications have been identified on all three NPs extracted from porcine heart and human prostate tissues. In addition, two *O*-glycosylation sites were identified on the mature ANP hormone, both within the highly conserved receptor binding region. Further *in vivo* and *in vitro* assays have demonstrated ANP glycosylation to positively affect circulating half-life by hampering the activity of the NPs degrading enzymes. However, ANP glycosylation negatively affected NPR-A activation. Interestingly, *O*-glycosylated proANP molecules were found in plasma extracted from human patients with a relatively high concentration of proBNP, estimated to account for 10% of all circulating proANP (146). Vodovar et al. (147) conducted studies on plasma obtained from 683 patients. It was revealed that HF patients had 1.5-fold higher concentrations of *O*-glycosylated proBNP compared to patients with ADHF or patients with dyspnea of non-cardiac origin. Furthermore, a significant negative correlation was observed between the concentration of glycosylated proBNP and the activation byproduct NT-proBNP, in both ADHF and non-ADHF patients. Moreover, despite having no difference in furin plasma concentrations, the enzyme’s activity was extremely high in ADHF patients among all three subgroups. Interestingly, no difference was observed in corin’s activity or concentration between all groups. These observations suggest that post-translational modifications likely occur in any case of chronic, but not acute, overproduction of proBNP.

Altogether, these data suggest an additional explanation for the reduced response to NPs resulted from the post-translational *O*-glycosylation of proBNP, which was shown to be enhanced in chronic HF and reduced in the acute state.

*Increase in peripheral degradation and NPR-C clearance*: The increase in NPs peripheral degradation and NPR-C clearance occurring in HF may also contribute to this phenomenon. Once released into the circulation, NPs may undergo proteolytic degradation by several enzymes. Neprilysin (NEP), dipeptidyl peptidase 4 (DPPIV), insulin-degrading enzyme (IDE), peptidyl arginine aldehyde protease (PAAP), and meprin-A are all proteolytic enzymes capable of degrading and inactivating the NPs, each having different cleavage site and distinct affinity to the various peptide forms (148–153). One possible explanation for the NPs paradox seen in HF may be the abundance of smaller and inactive NPs as a result of enzymatic degradation in the circulation (154, 155).

In a study published by Dos Santos et al. (156), HF patients exhibited a 130% increase in circulating DPPIV activity compared to healthy subjects, with an inverse correlation between the increase in enzyme activity and left ventricular ejection fraction (LVEF). Similar findings were observed in rats with HF compared to sham-operated animals, with the former demonstrating an increase in both the abundance and the activity of DDPIV, both in the plasma and in heart tissue (156). Furthermore, when treated with a DDPIV inhibitor for 6 weeks, HF-induced rats exhibited a significant attenuation of left ventricle end-diastolic pressure, systolic performance, chamber stiffness, cardiac remodeling, and pulmonary congestion.

In another study conducted by Bayes-Genis et al. (157), it was demonstrated that in patients with HF, circulating levels of NEP positively correlated with hospitalization and cardiovascular death. Interestingly, upregulation of mRNA and immunostaining of NEP in the kidneys of rats subjected to different HF models was evident (158). These findings were of paramount importance as they provided a scientific rational for the development of drugs aimed at targeting NEP for clinical use. Indeed, studies have shown that dual blockade of AT1R and NEP in HF patients was more efficient in reducing the mortality from cardiovascular causes or hospitalization due to worsening HF than was ACE inhibition alone (159, 160). Moreover, combined inhibition of AT1R and NEP led to a greater reduction in NT-proBNP serum levels in patients admitted with ADHF, as compared to ACE inhibitors alone (161). These data indicate that NEP plays a major role in HF syndrome and eliminating its action is beneficial in HF patients.

In addition to catalytic degradation, evidence suggests an increase in NPs clearance *via* NPR-C in patients with HF (158). As expected, blockade of the NPR-C in experimental HF induced a dose-dependent increase in ANP and cGMP plasma levels, as well as natriuresis and diuresis (162). These findings shed light on the adverse role of NPs clearance in HF, which along with NPs peripheral degradation contribute to the NPs paradox seen in HF.

Altogether, these data suggest that NPs degrading enzymes, including DPPIV and NEP, together with increased intracellular clearance, may play a crucial role in the development of NPs blunted response in HF.

*Downregulation of NPR-A receptors and changes in downstream signaling:* Downregulation of NPR-A receptors and changes in downstream signaling might play an important role in this state of hyporesponsiveness to NPs. The increase in NPs degradation and clearance may partially explain the NPs paradox in HF, especially the blunted response to the administration of synthetic active NPs seen in patients with HF (163). Several studies conducted on HF patients and HF models of experimental animals have suggested a downregulation of NPR-A activity and expression in different tissues, including the kidney (139). It should also be emphasized that elevated expression of phosphodiesterase-5 (PDE5), a cGMP hydrolyzing enzyme, might also contribute to renal hyporesponsiveness to NPs (139). These data shed more light on the complexity of HF syndrome and suggest that multiple mechanisms underlie the renal hypo-responsiveness to NPs in HF.

*Aberrant natriuretic peptides activation*: In addition to the abovementioned factors and mechanisms involved in the pathogenesis and the development of the NPs paradox in HF, the NPs machinery might constitute a major player in the evolvement of this pathological state. An aberrant machinery system unable to meet the body’s requirements in HF, in which it is incapable of producing and/or secreting mature and active hormones, may lead to a pathological state of NPs deficiency and a subsequent neurohormonal imbalance.

Corin is a type-II transmembrane serine protease expressed mainly in the heart (164, 165). By converting proANP and proBNP to their active forms through precise and regulated enzymatic cleavage (124, 125), corin is an important regulator of water and sodium balance, blood pressure, and cardiac remodeling. Therefore, disruption of its expression and/or activity may contribute to the development of several cardiovascular diseases (166–173).

Despite its crucial role, only a few experimental and clinical studies examined the status of cardiac corin under pathological conditions. Moreover, the reported findings were inconsistent. While some studies demonstrated upregulation of corin, others reported down-regulation of this enzyme in HF (168, 174–180). These conflicting results may stem from the application of different models of HF, the duration of HF, the studied chamber of the heart, and more. Yet, the demonstration of decreased cardiac levels of corin in HF may subsequently suggest poor and insufficient activation of NPs and thus partially explain the aberrant renal response to NPs. In this context, the functional role of cardiac corin in HFrEF was experimentally demonstrated by the genetic restoration of reduced cardiac corin levels in mice with DCM, where it caused improvement of contractile function, suppression of pleural edema, and extended lifespan through cleavage of the pro-ANP and cGMP modulation (168, 181). However, restoration of depressed cardiac corin expression improved systolic function and reduced HF-related systemic and pulmonary edema along with attenuation of HFrEF and survival prolongation through mechanism(s) independent from proANP cleavage (182).

Additionally, corin is also found in the blood system in its circulating forms. Previous studies demonstrated the decreased concentration of soluble circulating corin in patients with HF and acute myocardial infarction, compared to healthy individuals (183–191). Noteworthy, clinical studies demonstrated that decompensated heart failure, assessed by edema and elevated plasma ANP/BNP levels, is associated with reduced corin levels and decreased cleavage of proANP/proBNP peptides (179, 192–195). These observations suggest an inefficient activation of secreted pro-natriuretic peptides which conceivably contributes to HFrEF decompensation (191, 194, 195). In contrast to these findings, Wang et al. (192) reported that acute myocardial infarction (<72 h) induces elevated levels of circulating corin along with a decrease in cardiac corin levels. Interestingly, plasma corin levels were inversely correlated with heart function at the early phase of acute myocardial infarction, thus may reflect the severity of myocardial damage.

Moreover, corin is synthesized as an inactive zymogen and is subsequently activated by proprotein convertase subtilisin/kexin-6 (PCSK6) (196, 197). One might suggest that disturbance in the expression and/or activity of PCSK6 may lead to inactivated corin, and subsequently unprocessed and inactive natriuretic peptides. Recently, we demonstrated decreased cardiac PCSK6 expression and immunoreactive levels in rats with decompensated HF (198). To the best of our knowledge, up to date, there is still no additional data concerning PCSK6 cardiac and circulating abundance or activity in HF, and its contribution to cardiac remodeling, corin activation, and natriuretic peptide processing in this context is unknown.

*Inaccurate immunoassays*: An additional contributing factor may be the increase in the secretion of the less active forms of BNP seen in HF patients, rather than the mature active hormone BNP_1–32_. These peptides include NT-proBNP_1–76_, proBNP_1–108_, and additional small peptides resulting from peripheral enzymatic degradation and include BNP_3–32_, BNP_5–32_, and BNP_8–32_ (199–202). Today, different commercial immunoassays are available for detecting NT-proBNP_1–76_ and BNP_1–32_ in human plasma. Importantly, these assays can also detect other less-active forms of BNP, whether as a result of degradation or glycosylation, and their specificity and sensitivity depend on the cross-reactivity of each assay (155).

A study by Hawkridge et al. (203) measured the active BNP_1–32_ levels in the plasma of four HF patients using mass spectrometry. Surprisingly, the group did not detect BNP_1–32_ in these plasma despite the substantially high levels of BNP_1–32_ reported by using commercial immunoassay on the same plasma. An additional study by Seferian et al. (204) revealed that proBNP_1–108_ is the major immunoreactive BNP form in plasma of HF patients, by using specific monoclonal antibodies.

Thus, the high circulating-BNP-levels state seen in HF can be misleading, as these elevated levels may primarily reflect both the inactive and the less potent forms of NPs. In this case, while high circulating BNP levels may constitute a reliable biomarker for HF, patients may be in a state of natriuretic peptide deficiency.

### Renal venous congestion

The observation that elevated CVP results in increased outflow pressure in the renal veins in association with renal dysfunction has been first recognized in 1931 (205). In heart failure patients, several studies showed an association between increased CVP and renal dysfunction (32, 206) (Figure 2).

The contribution of renal venous congestion to renal dysfunction in HF is complex, involving multiple contributing mechanisms (Figure 2) (7). Increased renal venous pressure increases pressures along the renal vascular tree, thus decreasing RBF (207) and arteriovenous pressure gradient in the glomerulus and thereby decreasing GFR (7).

An increase in renal venous pressure can elevate intrarenal interstitial pressure, which in turn affects the entire capillary bed and the tubules (208–211). The kidney is an encapsulated organ and therefore responds to raised renal venous pressures with a disproportionate elevation in intracapsular pressure, which also leads to increased intrarenal interstitial pressure (212). This leads to compression of the tubules (with relative sparing of the renal cortex), increased tubular fluid pressure, with reduced GFR due to an increase in hydrostatic pressure in Bowman’s capsule (210). Increased interstitial pressure may promote tubular inflammation and fibrosis, affect tubuloglomerular feedback and activate neurohormonal systems (213) (Figure 2).

However, the pattern of renal venous pressure increase in patients with heart failure is substantially different from those used in experimental models, where renal venous pressure was abruptly raised to extremely high values that are usually not seen even in patients with severe heart failure (e.g., 25–50 mm Hg) (209, 210, 213, 214). For example, in the isolated perfused rat kidney model, GFR was not significantly altered until the imposed venous pressure reached 25 mm Hg (210). In a dog model of renal vein hypertension, renal dysfunction occurred only when cardiac output was concomitantly reduced (24). Notwithstanding, it is possible that the kidneys are more sensitive to elevated CVP in the setting of chronic heart failure, such that GFR may fall with lower CVP than required in healthy animals (215).

In experimental models, renal dysfunction secondary to venous congestion is potentially reversible, at least partially. Lowering renal vein pressure immediately improved its associated renal hemodynamic derangements, leading to improved urine output and GFR. Clinically, the potential for reversibility is demonstrated in patients who show improved renal function after decongestive therapy with diuretics or patients with improvement in right ventricular function secondary to a reduction in pulmonary pressures (216–218).

Mullens et al. were the first to report that elevated CVP was associated with WRF in severe HF with reduced cardiac index who required inotropes or vasodilators (32). In this study, the association between baseline venous congestion and worsening renal function was stronger than the association with CO.

However, the findings of this initial report were not consistent. In subsequent studies, the magnitude of reduction in CVP did not result in improvement in renal function or lower incidence of WRF (219–221).

The effect of the reduction in venous pressure may be difficult to demonstrate clinically given that pressure has little correlation with volume in the venous system (222). Veins have a high compliance and are easily able to accommodate changes in blood volume. The compliant nature of the venous vessels (which contain >70% of total blood volume) establishes a relative pressure-volume disconnection, allowing large changes in blood volume to be associated with small changes in pressure. Thus, even an effective treatment of volume overload may not be sufficient to produce a meaningful reduction in CVP and, in turn, in renal function.

More recent attempts to prove this concept clinically in patients with HF involve a device-based direct reduction (rather than with diuretics and vasodilators) of renal vein pressure. Revamp Medical developed the percutaneous Doraya catheter, which is positioned infra-renally (223). The distal frame opening can be adjusted to produce a partial obstruction of the venous flow at this level, thus resulting in a reduction of renal venous pressure. The first-in-human study of the Doraya catheter in acute heart failure patients (NCT03234647) demonstrated a substantial pressure reduction at the level of the renal veins (12.4 ± 4.7 mm Hg compared to baseline). This was associated with an increase in urine output from 77.1 ± 25 mL/h at baseline, to 200.8 ± 93 mL/h during device deployment on a stable diuretic dose (223).

Magenta Medical developed a transcatheter renal venous decongestion system designed to reduce the pressure in both renal veins using an axial-flow pump-head positioned in the inferior vena cava (IVC). Two sealing elements are positioned above and below the kidneys to compartmentalize the renal segment of the IVC and allow selective reduction of renal venous pressures. A clinical trial (NCT03621436) is underway.

### Intra-abdominal pressure

The renal consequences of intra-abdominal hypertension (IAH) secondary to fluid overload and visceral edema, and its association with acute kidney injury (AKI), have been first recognized in critically ill patients such as those with abdominal surgery, trauma, and major burns (7, 224, 225). More recently, this entity has been implicated as an important contributor to renal dysfunction in HF based on animal (215, 226) and human (227) studies.

The normal intra-abdominal pressure (IAP) ranges from 4 to 7 mmHg (228). IAH is defined as a sustained or repeated abnormal increase of IAP to ≥12 mmHg (224, 228). However, given the susceptibility to renal dysfunction in heart failure, there are data suggesting that even smaller increases in IAP, in the range of 8–12 mm Hg, can induce a reduction in GFR and sodium excretion (215, 229).

The deleterious effects of IAP on the kidney are closely linked and overlap with those of the aforementioned pathophysiology of venous congestion (Figure 2) and congestive nephropathy (227). There is a direct compression of abdominal contents that result in a prominent reduction in RPF (compression of renal arteries) and elevation in renal parenchymal and renal vein pressures (228). The abdominal perfusion pressure (APP) is defined as the difference between the mean arterial pressure and the IAP. As IAP increases, the perfusion of organs or vessels in or near the abdomen falls even with normal mean arterial pressure (224).

In normal physiologic states, hydrostatic pressure in Bowman’s space (and therefore in the proximal tubules) is negligible, promoting glomerular filtration; in the presence of IAH, Bowman’s space and proximal tubular pressure will be increased close to IAP, resulting in reduced GFR (214, 224, 230). The decreased glomerular hydrostatic pressure (due to hypoperfusion) also contributes to the reduction in the glomerular filtration gradient (225). Elevated IAP also up-regulates the RAAS (231). Because IAH increases renal venous pressure, which in turn produces renal interstitial congestion, the result is a vicious cycle that further increases IAP and renal venous pressure (224).

A reduction in IAP with paracentesis leading to improvement in kidney function has been reported in hepatorenal syndrome (224). Because the majority of patients with IAH are hypervolemic, systemic volume removal also leads to a prompt decrease in IAP and concomitantly improves renal venous hypertension (232).

Few data are available on IAH in HF. A study of 40 patients has shown that 24 (60%) had elevated IAP and 4 (10%) demonstrated IAH despite the absence of overt ascites (229). A higher prevalence of impaired renal function was observed in patients with IAP, and improvement in kidney function was associated with a reduction of IAP. In a small prospective analysis of patients with acute heart failure, diuretic resistance, and mild IAH, a reduction in IAP with ultrafiltration or paracentesis (if ascites were present) resulted in an increase in urine output and a reduction in serum creatinine (227).

Currently, few data are available regarding the indications to measure IAP in patients with HF and fluid overload. If IAH is documented, prompt volume removal must be considered to decrease IAP.

## Edema therapy in heart failure

Congestion is also a hallmark of HFpEF and is associated with adverse outcomes (233, 234). Patients with HFpEF and HFrEF present acutely with comparable clinical and echocardiographic evidence of venous congestion and renal dysfunction (235).

In addition to salt and fluid retention, congestion can also be triggered by fluid redistribution (236, 237). Blood redistribution across different compartments may lead to rapid changes in systemic and pulmonary venous pressures despite constant total blood volume (238, 239). For example, sympathetic activation can increase preload by a functional shift of blood from the splanchnic venous reservoir to the central vascular compartment (222). A rapid increase in systemic pressure and systemic vascular resistance, leading to afterload mismatch may also trigger symptoms in patients with excessive afterload sensitivity and impaired preload reserve (240).

**FIGURE 3:**
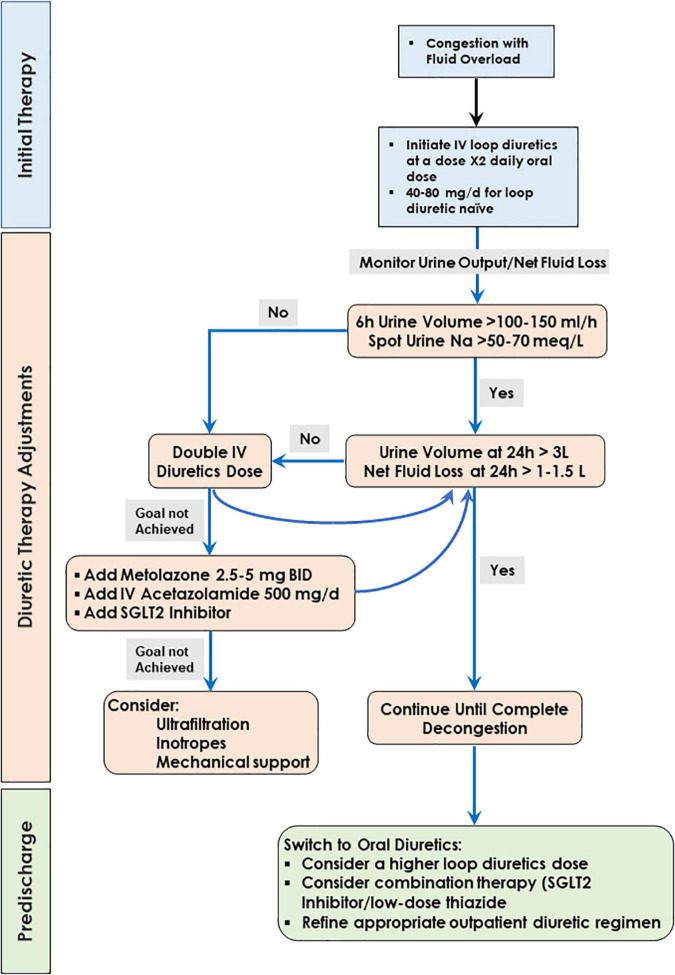
Algorithm for decongestive therapy.

Current recommendations for the treatment of hospitalized patients with fluid overload (241–243) adopt an algorithm originally used in the CARRESS-HF trial (244) (Figure 3). The algorithm entails a stepped pharmacologic care that ensures appropriate diuretic doses, with frequent monitoring of urine output and clinical response (Figure 3) but has not been rigorously validated in clinical trials.

### Diuretics

The efficacy of the diuretic effect is assessed by measuring urine output and spot sodium urine concentration, where effective diuresis is defined as urine volume of >100 to 150 mL/h or urine spot Na+ >50–70 mEq/L with a urine output goal of 3–5 L/day (241). Of note, fluid intake during decongestive therapy can be substantial, such that net fluid loss remains small despite apparently satisfactory urine output. In one study, fluid intake exceeded 50% of urine output in two-thirds of the patients (245). In addition, the patient’s weight is not considered in these protocols. A urine output of 2 L/day can be interpreted differently in a patient weighing 70 or 110 Kg.

When this goal is not met, doubling the diuretic dose is recommended. Because the dose-response relationship of a loop diuretic is log-linear, the natriuretic response to each double the dose of a loop diuretic may be modest (246, 247). The addition of oral metolazone or chlorothiazide may provide a greater natriuretic response and urine output (248, 249), with an increased risk for hypokalemia, hyponatremia, worsening renal function, and hypotension (249). The addition of acetazolamide to loop diuretic therapy in patients with acute decompensated heart failure resulted in a higher incidence of successful decongestion (250).

Recently, sodium-glucose co-transporter 2 (SGLT2) inhibitors have been shown to induce significant natriuresis, particularly when added to loop diuretics (251–253).

The usual goal is complete decongestion, with the absence of signs and symptoms of elevated resting filling pressures, because rehospitalization rates and mortality are considerably lower in patients who are free of clinical congestion at the time of hospital discharge (241, 242). However, complete decongestion can be hampered by several factors including low cardiac output, dominant right heart failure and severe pulmonary hypertension, severe renal dysfunction, low plasma oncotic pressure, and symptomatic hypotension (242). For these patients, the goal of edema resolution may need to be relaxed. For example, patients with right ventricular dysfunction, pulmonary hypertension, and tricuspid regurgitation often cannot be decongested to a normal jugular venous pressure. Currently, there is a great interest in novel clinical approaches to treat decongestion. Ongoing clinical trials of drugs or devices for the treatment of congestion are depicted in Table 1.

**TABLE 1:** Ongoing clinical trials of drugs or devices for the treatment of congestion in HF.

Study	ClinicalTrials.gov identifier	Agent/Device	Clinical setting	Hypothesis
ADVOR trial (254)	NCT03505788	Acetazolamide	AHF	Acetazolamide improves decongestion when combined with loop diuretic therapy in AHF
AVANTI trial (255)	NCT03901726	Pecavaptan, a dual V1a/V2 AVP receptors antagonist	AHF	Pecavaptan improves decongestion when combined with loop diuretic therapy in AHF
TRANSFORM-HF trial (256)	NCT03296813	Torsemide	Stable HF	Torsemide comparative-effectiveness trial of torsemide versus furosemide
REVERSE-HF	NCT05318105	Ultrafiltration – Aquadex system	AHF	Ultrafiltration versus IV diuretics in worsening heart failure
DICTATE-AHF trial (257)	NCT04298229	Dapagliflozin	AHF	Efficacy and safety of initiating dapagliflozin within the first 24 h of hospitalization in patients with AHF compared to usual care
Reprieve cardiovascular system (258)	NCT05015764	Reprieve system	AHF	Reprieve system, which continuously monitors urine output and delivers a matched volume of hydration fluid sufficient to maintain the set fluid balance rate, compared with standard diuretic-based regimen improves decongestion in AHF
Aortix CRS pilot study	NCT04145635	Aortix pump	Cardiorenal syndrome	An elevation of the safety and performance of the Aortix system for intra-aortic mechanical circulatory support in patients with cardiorenal syndrome
SAHARA study	NCT04882358	Alfapump DSR system	Volume overloaded HF	Feasibility and safety study of the alfapump DSR system in the treatment of volume overloaded heart failure
DAPA ACT HF-TIMI 68	NCT04363697	Dapagliflozin	AHF	Effect of in-hospital initiation of dapagliflozin versus placebo on the clinical outcome of cardiovascular death or worsening heart failure
RELIEVE-HF trial (259)	NCT03499236	V-Wave Interatrial Shunt	Stable HF	Safety and effectiveness of the V-Wave Interatrial Shunt System for improving meaningful clinical outcomes in patients with NYHA functional class II, III, or ambulatory class IV HF

### Renin-angiotensin-aldosterone system inhibitors

Renin-angiotensin-aldosterone system inhibitors significantly improved morbidity and mortality in chronic HF patients with HFrEF (260). The beneficial effects of RAAS blockers in HFrEF occur at the cardiac and vasculature levels, yet they induce a reduction in GFR and WRF due to efferent vasodilation, thus increasing the risk of poor clinical outcomes although the mortality benefit is maintained (53, 89, 261). Evidence for the involvement of the RAAS in the development of elevated sodium balance can be derived from studies showing that the renal and hemodynamic response to ANP is impaired in experimental CHF of various etiologies, and that administration of either ARB or ACE inhibitor restores this blunted response to ANP (13). In the last two decades, the adverse role of aldosterone in the pathogenesis of CHF was established (262). Besides promoting sodium retention, aldosterone contributes to vascular and cardiac remodeling by inducing perivascular and interstitial fibrosis (262, 263). Therefore, the addition of small doses of spironolactone or finerenone to standard therapy substantially reduces the mortality rate and morbidity in CHF patients (262, 264, 265). At the renal level, RAAS blockers induce diuretic and natriuretic responses, especially at the initial stages of their administration (266).

### Beta-blockers

When given with ACEi or diuretics, β-blockers have been shown to reduce mortality and morbidity in patients with HFrEF (267). Beta-blockers should be initiated in clinically stable, euvolemic patients at a low dose and gradually increased to the maximal tolerated dose. Since activation of the β1 receptor stimulates renin secretion, one may assume that the beneficial effects of β-blockers at both the cardiac and renal levels are partially attributed to attenuation of renin secretion.

### Sodium-glucose co-transporter 2 inhibitor

Several clinical trials have demonstrated that SGLT2 inhibitors cause a significant reduction in HF hospitalization (268–270). These beneficial effects on HFrEF patients persist even in non-diabetic patients as was reported by DAPA-HF and EMPEROR-Reduced trials (271–273). Specifically, dapagliflozin significantly reduced the primary endpoint of worsening HF or cardiovascular death in the non-diabetic and diabetic groups, respectively, showing its similar efficacy regardless of the presence or absence of diabetes (273). Similar results were obtained by The EMPEROR-Reduced trial, where empagliflozin reduced the combined primary endpoint of cardiovascular death or HF hospitalization by 25% (272). In both DAPA-HF and EMPEROR-Reduced trials, attenuation of eGFR decline was observed following its greater initial drop due to the reduced glomerular hyperfiltration. The mechanisms underlying these beneficial effects include improvement in insulin secretion and sensitivity, osmotic diuretic and natriuretic effects, and resulted reduction of preload and afterload, augmentation of loop diuretics natriuretic action, improvement in myocardial energetics, increase oxygen delivery to the failing myocardium secondary to hemoconcentration, anti-oxidative stress and anti-inflammation (268, 274). At the renal level, SGLT2 inhibitors exert cardiorenal protection beyond these effects, where their natriuretic action due to inhibition of SGLT2 at the proximal tubule and increased sodium to macula densa activates tubuleoglomerular feedback (TGF) as evident by afferent arteriole vasoconstriction, thus preserving renal function as well as improve renal outcomes observed in patients with HF (268, 274).

### Sacubitril/valsartan

Since either ARBs or neprilysin inhibitors have shown to improve cardiac and renal function in both experimental and clinical CHF (1, 268, 275), a combined drug, sacubitril/valsartan (ARNI) was introduced in the last decade and became a key drug for the treatment of HFrEF (276, 277). Recent data demonstrated that sacubitril/valsartan, a combined angiotensin receptor blocker and neprilysin inhibitor, significantly reduced cardiovascular mortality and hospitalization due to worsening HF among HFrEF patients, as compared to an ACEi (160). Besides counteracting the negative cardiorenal effects of the upregulated RAAS *via* AT1R blockade, inhibition of neprilysin by ARNI increases NPs by preventing their degradation, thus inducing natriuresis, reduction of blood pressure, inhibition of cardiac myocyte hypertrophy, apoptosis, and fibrosis (277).

## Summary and conclusion

HFrEF is associated with renal dysfunction, reflecting the interconnection between the heart and the kidney. Multiple mechanisms underlie this interdependence including hemodynamic alterations manifested by insufficient peripheral and renal perfusion, along with activation of neurohormonal systems. Exaggerated activation of these factors results in deleterious effects on both the kidneys and the heart, including sodium and water retention, vasoconstriction, increased central and renal venous hypertension/congestion, as well as increased IAP. The latter was shown to induce renal hypoperfusion and hypofiltration. Besides the activation of vasoconstrictor/anti-natriuretic neurohormonal systems, HF is elevated by levels of NPs, yet their beneficial natriuretic and anti-fibrotic effects are attenuated due to the supremity of the deleterious neurohormonal systems as evident by persistent sodium and water retention and cardiomyopathy. As our understanding of the pathogenesis of cardiac remodeling and sodium retention characterizing CHF is gradually improving, the introduction of mechanistic-based treatments equivalently increases (243). These include neurohumoral blockers such as β-receptor blockers, ACE inhibitors, ARBs, aldosterone receptor antagonists, besides diuretics, the cornerstone therapy, and most recently SGLT2 inhibitors. All these therapies aimed at reducing cardiac remodeling and restoring normal sodium balance along the euvolemic state (88, 267, 278). Although the application of diuretic therapy is widely adopted in acute decompensated CHF and chronic HF, the need for loop diuretic utilization should be tapered continuously to prevent the rebound of sodium retaining/vasoconstrictor systems. Intriguingly, new drugs in the treatment of chronic HF, which also decrease sodium avidity, have demonstrated improved HF hospitalizations and survival (4). Therefore, the introduction of SGLT2 inhibitors and widening their clinical use beyond diabetes may represent a game changer in the treatment of HFrEF and renal dysfunction.

## Author contributions

ZA, EK, TK, and DA drafted the manuscript. ZA, EK, and DA prepared the figures. All authors edited the manuscript and provided constructive comments.

## References

[1] Skorecki K Winaver J Abassi Z. *Brenner and Rector’s The Kidney.* 8th ed. In: Brenner B Rector F editors. Amsterdam: Elsevier (2008). p. 398–458.

[2] Mazurek JA Jessup M. Understanding heart failure. *Heart Fail Clin.* (2017) 13:1–19. 10.1016/j.hfc.2016.07.001 27886916

[3] Metra M Teerlink JR. Heart failure. *Lancet.* (2017) 390:1981–95. 10.1016/S0140-6736(17)31071-1 28460827

[4] Mullens W Verbrugge FH Nijst P Tang WHW. Renal sodium avidity in heart failure: from pathophysiology to treatment strategies. *Eur Heart J.* (2017) 38:1872–82. 10.1093/eurheartj/ehx035 28329085

[5] Chaney E Shaw A. Pathophysiology of fluid retention in heart failure. *Contrib Nephrol.* (2010) 164:46–53. 10.1159/000313720 20427993

[6] Schefold JC Filippatos G Hasenfuss G Anker SD von Haehling S. Heart failure and kidney dysfunction: epidemiology, mechanisms and management. *Nat Rev Nephrol.* (2016) 12:610–23. 10.1038/nrneph.2016.113 27573728

[7] Aronson D. Cardiorenal syndrome in acute decompensated heart failure. *Expert Rev Cardiovasc Ther.* (2012) 10:177–89. 10.1586/erc.11.193 22292874

[8] Kazory A Elkayam U. Cardiorenal interactions in acute decompensated heart failure: contemporary concepts facing emerging controversies. *J Card Fail.* (2014) 20:1004–11. 10.1016/j.cardfail.2014.09.005 25230240

[9] Virzì GM Clementi A Brocca A de Cal M Vescovo G Granata A The hemodynamic and nonhemodynamic crosstalk in cardiorenal syndrome type 1. *Cardiorenal Med.* (2014) 4:103–12. 10.1159/000362650 25254032 PMC4164059

[10] Schrier RW. Pathogenesis of sodium and water retention in high-output and low-output cardiac failure, nephrotic syndrome, cirrhosis, and pregnancy (1). *N Engl J Med.* (1988) 319:1065–72. 10.1056/NEJM198810203191606 3050518

[11] Schrier RW. Body fluid volume regulation in health and disease: a unifying hypothesis. *Ann Intern Med.* (1990) 113:155–9. 10.7326/0003-4819-113-2-155 2193561

[12] Schrier RW. A unifying hypothesis of body fluid volume regulation. The Lilly lecture 1992. *J R Coll Physicians Lond.* (1992) 26:295–306. 1404027 PMC5375453

[13] Abassi Z Goltsman I Karram T Winaver J Hoffman A. Aortocaval fistula in rat: a unique model of volume-overload congestive heart failure and cardiac hypertrophy. *J Biomed Biotechnol.* (2011) 2011:729497. 10.1155/2011/729497 21274403 PMC3025398

[14] Pugliese NR Masi S Taddei S. The renin-angiotensin-aldosterone system: a crossroad from arterial hypertension to heart failure. *Heart Fail Rev.* (2020) 25:31–42. 10.1007/s10741-019-09855-5 31512149

[15] Haraldsson B. Physiological studies of macromolecular transport across capillary walls. Studies on continuous capillaries in rat skeletal muscle. *Acta Physiol Scand Suppl.* (1986) 553:1–40. 3466511

[16] Magrini F Niarchos AP. Hemodynamic effects of massive peripheral edema. *Am Heart J.* (1983) 105:90–7. 10.1016/0002-8703(83)90283-1 6849245

[17] Brace RA Guyton AC. Effect of hindlimb isolation procedure on isogravimetric capillary pressure and transcapillary fluid dynamics in dogs. *Circ Res.* (1976) 38:192–6. 10.1161/01.res.38.3.192 1248067

[18] Epstein FH Post RS Mcdowell M. The effects of an arteriovenous fistula on renal hemodynamics and electrolyte excretion. *J Clin Invest.* (1953) 32:233–41. 10.1172/JCI102732 13044832 PMC438334

[19] Starling E. Physiological factors involved in the causation of dropsy. *Lancet.* (1896) 147:1407–10.

[20] Harrison T. The pathogenesis of congestive heart failure. *Medicine.* (1935) 14:255.

[21] Stead EA Jr Ebert RV. Shock syndrome produced by failure of the heaRT. *Arch Intern Med.* (1942) 69:369–83. 10.1001/archinte.1942.00200150002001

[22] Peters JP. The role of sodium in the production of edema. *N Engl J Med.* (1948) 239:353–62. 10.1056/NEJM194809022391001 18879457

[23] Borst JGG de Vries LA. The three types of “natural” diuresis. *Lancet.* (1950) 2:1–6. 10.1016/s0140-6736(50)91818-6 15437880

[24] Priebe HJ Heimann JC Hedley-Whyte J. Effects of renal and hepatic venous congestion on renal function in the presence of low and normal cardiac output in dogs. *Circ Res.* (1980) 47:883–90. 10.1161/01.res.47.6.883 7192184

[25] Zucker IH Wang W Brändle M Schultz HD Patel KP. Neural regulation of sympathetic nerve activity in heart failure. *Prog Cardiovasc Dis.* (1995) 37:397–414. 10.1016/s0033-0620(05)80020-9 7777669

[26] Thames MD Kinugawa T Smith ML Dibner-Dunlap ME. Abnormalities of baroreflex control in heart failure. *J Am Coll Cardiol.* (1993) 22:56A–60A. 10.1016/0735-1097(93)90464-c 8104206

[27] Gabrielsen A Bie P Holstein-Rathlou NH Christensen NJ Warberg J Dige-Petersen H Neuroendocrine and renal effects of intravascular volume expansion in compensated heart failure. *Am J Physiol Regul Integr Comp Physiol.* (2001) 281:R459–67. 10.1152/ajpregu.2001.281.2.R459 11448848

[28] DiBona GF Herman PJ Sawin LL. Neural control of renal function in edema-forming states. *Am J Physiol.* (1988) 254:R1017–24. 10.1152/ajpregu.1988.254.6.R1017 3381907

[29] DiBona GF Sawin LL. Reflex regulation of renal nerve activity in cardiac failure. *Am J Physiol.* (1994) 266:R27–39. 10.1152/ajpregu.1994.266.1.R27 8304550

[30] Ichikawa I Pfeffer JM Pfeffer MA Hostetter TH Brenner BM. Role of angiotensin II in the altered renal function of congestive heart failure. *Circ Res.* (1984) 55:669–75. 10.1161/01.res.55.5.669 6091942

[31] Nishikimi T Frohlich ED. Glomerular hemodynamics in aortocaval fistula rats: role of renin-angiotensin system. *Am J Physiol.* (1993) 264:R681–6. 10.1152/ajpregu.1993.264.4.R681 8476110

[32] Mullens W Abrahams Z Francis GS Sokos G Taylor DO Starling RC Importance of venous congestion for worsening of renal function in advanced decompensated heart failure. *J Am Coll Cardiol.* (2009) 53:589–96. 10.1016/j.jacc.2008.05.068 19215833 PMC2856960

[33] Volpe M Carnovali M Mastromarino V. The natriuretic peptides system in the pathophysiology of heart failure: from molecular basis to treatment. *Clin Sci.* (2016) 130:57–77. 10.1042/CS20150469 26637405 PMC5233571

[34] Mentz RJ O’Connor CM. Pathophysiology and clinical evaluation of acute heart failure. *Nat Rev Cardiol.* (2016) 13:28–35. 10.1038/nrcardio.2015.134 26370473

[35] Rosner MH Ronco C Okusa MD. The role of inflammation in the cardio-renal syndrome: a focus on cytokines and inflammatory mediators. *Semin Nephrol.* (2012) 32:70–8. 10.1016/j.semnephrol.2011.11.010 22365165

[36] Gottschalk CW Mylle M. Micropuncture study of pressures in proximal tubules and peritubular capillaries of the rat kidney and their relation to ureteral and renal venous pressures. *Am J Physiol.* (1956) 185:430–9. 10.1152/ajplegacy.1956.185.2.430 13327061

[37] Lebrie SJ Mayerson HS. Influence of elevated venous pressure on flow and composition of renal lymph. *Am J Physiol.* (1960) 198:1037–40. 10.1152/ajplegacy.1960.198.5.1037 14415049

[38] Hillege HL Girbes AR de Kam PJ Boomsma F de Zeeuw D Charlesworth A Renal function, neurohormonal activation, and survival in patients with chronic heart failure. *Circulation.* (2000) 102:203–10. 10.1161/01.cir.102.2.203 10889132

[39] Bell NH Schedl HP Bartter FC. An explanation for abnormal water retention and hypoosmolality in congestive heart failure. *Am J Med.* (1964) 36:351–60. 10.1016/0002-9343(64)90161-5 14131879

[40] Bennett WM Bagby GCJ Antonovic JN Porter GA. Influence of volume expansion on proximal tubular sodium reabsorption in congestive heart failure. *Am Heart J.* (1973) 85:55–64. 10.1016/0002-8703(73)90525-5 4682006

[41] Johnston CI Davis JO Robb CA Mackenzie JW. Plasma renin in chronic experimental heart failure and during renal sodium “escape” from mineralocorticoids. *Circ Res.* (1968) 22:113–25. 10.1161/01.res.22.2.113 5639033

[42] Schneider EG Dresser TP Lynch RE Knox FG. Sodium reabsorption by proximal tubule of dogs with experimental heart failure. *Am J Physiol.* (1971) 220:952–7. 10.1152/ajplegacy.1971.220.4.952 5551151

[43] Stumpe KO Sölle H Klein H Krück F. Mechanism of sodium and water retention in rats with experimental heart failure. *Kidney Int.* (1973) 4:309–17. 10.1038/ki.1973.122 4762578

[44] Mandin H Davidman M. Renal function in dogs with acute cardiac tamponade. *Am J Physiol.* (1978) 234:F117–22. 10.1152/ajprenal.1978.234.2.F117 623301

[45] Auld RB Alexander EA Levinsky NG. Proximal tubular function in dogs with thoracic caval constriction. *J Clin Invest.* (1971) 50:2150–8. 10.1172/JCI106709 5116206 PMC292149

[46] Levy M. Effects of acute volume expansion and altered hemodynamics on renal tubular function in chronic caval dogs. *J Clin Invest.* (1972) 51:922–38. 10.1172/JCI106887 5014619 PMC302206

[47] Park F Mattson DL Skelton MM Cowley AWJ. Localization of the vasopressin V1a and V2 receptors within the renal cortical and medullary circulation. *Am J Physiol.* (1997) 273:R243–51. 10.1152/ajpregu.1997.273.1.R243 9249556

[48] Costanzo MR Jessup M. Treatment of congestion in heart failure with diuretics and extracorporeal therapies: effects on symptoms, renal function, and prognosis. *Heart Fail Rev.* (2012) 17:313–24. 10.1007/s10741-011-9248-0 21559880

[49] Metra M Davison B Bettari L Sun H Edwards C Lazzarini V Is worsening renal function an ominous prognostic sign in patients with acute heart failure? The role of congestion and its interaction with renal function. *Circ Heart Fail.* (2012) 5:54–62. 10.1161/CIRCHEARTFAILURE.111.963413 22167320

[50] Heywood JT Fonarow GC Costanzo MR Mathur VS Wigneswaran JR Wynne J. High prevalence of renal dysfunction and its impact on outcome in 118,465 patients hospitalized with acute decompensated heart failure: a report from the ADHERE database. *J Card Fail.* (2007) 13:422–30. 10.1016/j.cardfail.2007.03.011 17675055

[51] Ezekowitz J McAlister FA Humphries KH Norris CM Tonelli M Ghali WA The association among renal insufficiency, pharmacotherapy, and outcomes in 6,427 patients with heart failure and coronary artery disease. *J Am Coll Cardiol.* (2004) 44:1587–92. 10.1016/j.jacc.2004.06.072 15489090

[52] Hebert K Dias A Delgado MC Franco E Tamariz L Steen D Epidemiology and survival of the five stages of chronic kidney disease in a systolic heart failure population. *Eur J Heart Fail.* (2010) 12:861–5. 10.1093/eurjhf/hfq077 20484366

[53] Damman K Valente MAE Voors AA O’Connor CM van Veldhuisen DJ Hillege HL. Renal impairment, worsening renal function, and outcome in patients with heart failure: an updated meta-analysis. *Eur Heart J.* (2014) 35:455–69. 10.1093/eurheartj/eht386 24164864

[54] Testani JM Brisco-Bacik MA. Worsening renal function and mortality in heart failure: causality or confounding? *Circ Heart Fail.* (2017) 10:e003835. 10.1161/CIRCHEARTFAILURE.117.003835 28209768 PMC5382499

[55] Harrison-Bernard LM. The renal renin-angiotensin system. *Adv Physiol Educ.* (2009) 33:270–4. 10.1152/advan.00049.2009 19948673

[56] Brewster UC Setaro JF Perazella MA. The renin-angiotensin-aldosterone system: cardiorenal effects and implications for renal and cardiovascular disease states. *Am J Med Sci.* (2003) 326:15–24. 10.1097/00000441-200307000-00003 12861121

[57] Santos RAS Oudit GY Verano-Braga T Canta G Steckelings UM Bader M. The renin-angiotensin system: going beyond the classical paradigms. *Am J Physiol Heart Circ Physiol.* (2019) 316:H958–70. 10.1152/ajpheart.00723.2018 30707614 PMC7191626

[58] Basso N Terragno NA. History about the discovery of the renin-angiotensin system. *Hypertension.* (2001) 38:1246–9. 10.1161/hy1201.101214 11751697

[59] Sharma N Anders H-J Gaikwad AB. Fiend and friend in the renin angiotensin system: an insight on acute kidney injury. *Biomed Pharmacother.* (2019) 110:764–74. 10.1016/j.biopha.2018.12.018 30554115

[60] Forrester SJ Booz GW Sigmund CD Coffman TM Kawai T Rizzo V Angiotensin II signal transduction: an update on mechanisms of physiology and pathophysiology. *Physiol Rev.* (2018) 98:1627–738. 10.1152/physrev.00038.2017 29873596 PMC6335102

[61] Tigerstedt R Bergman PQ. Niere und kreislauf. *Skandinavisches Archiv Für Physiologie.* (1898) 8:223–71. 10.1111/j.1748-1716.1898.tb00272.x

[62] Danser AH Saris JJ Schuijt MP van Kats JP. Is there a local renin-angiotensin system in the heart? *Cardiovasc Res.* (1999) 44:252–65. 10.1016/s0008-6363(99)00202-3 10690302

[63] Sullivan RD Mehta RM Tripathi R Reed GL Gladysheva IP. Renin activity in heart failure with reduced systolic function-new insights. *Int J Mol Sci.* (2019) 20:3182. 10.3390/ijms20133182 31261774 PMC6651297

[64] Pieruzzi F Abassi ZA Keiser HR. Expression of renin-angiotensin system components in the heart, kidneys, and lungs of rats with experimental heart failure. *Circulation.* (1995) 92:3105–12. 10.1161/01.cir.92.10.3105 7586282

[65] Silver SA Beaubien-Souligny W Shah PS Harel S Blum D Kishibe T The prevalence of acute kidney injury in patients hospitalized with COVID-19 infection: a systematic review and meta-analysis. *Kidney Med.* (2021) 3:83–98.e1. 10.1016/j.xkme.2020.11.008 33319190 PMC7723763

[66] Brenner BM Cooper ME de Zeeuw D Keane WF Mitch WE Parving HH Effects of losartan on renal and cardiovascular outcomes in patients with type 2 diabetes and nephropathy. *N Engl J Med.* (2001) 345:861–9. 10.1056/NEJMoa011161 11565518

[67] Yusuf S Pitt B Davis CE Hood WB Cohn JN. Effect of enalapril on survival in patients with reduced left ventricular ejection fractions and congestive heart failure. *N Engl J Med.* (1991) 325:293–302. 10.1056/NEJM199108013250501 2057034

[68] Yusuf S Pitt B Davis CE Hood WBJ Cohn JN. Effect of enalapril on mortality and the development of heart failure in asymptomatic patients with reduced left ventricular ejection fractions. *N Engl J Med.* (1992) 327:685–91. 10.1056/NEJM199209033271003 1463530

[69] International Society of Nephrology. Summary of recommendation statements. *Kidney Int Suppl.* (2012) 2:341–2. 10.1038/kisup.2012.50 25018959 PMC4089720

[70] International Society of Nephrology. Summary of recommendation statements. *Kidney Int Suppl.* (2013) 3:5–14. 10.1038/kisup.2012.77 25598998 PMC4284512

[71] Weir MR Lakkis JI Jaar B Rocco MV Choi MJ Kramer HJ Use of renin-angiotensin system blockade in advanced CKD: an NKF-KDOQI controversies report. *Am J kidney Dis Off J Natl Kidney Found.* (2018) 72:873–84. 10.1053/j.ajkd.2018.06.010 30201547

[72] Maschio G Alberti D Janin G Locatelli F Mann JF Motolese M Effect of the angiotensin-converting-enzyme inhibitor benazepril on the progression of chronic renal insufficiency. The angiotensin-converting-enzyme inhibition in progressive renal insufficiency study group. *N Engl J Med.* (1996) 334:939–45. 10.1056/NEJM199604113341502 8596594

[73] Linz W Wiemer G Schaper J Zimmermann R Nagasawa K Gohlke P Angiotensin converting enzyme inhibitors, left ventricular hypertrophy and fibrosis. *Mol Cell Biochem.* (1995) 147:89–97. 10.1007/BF00944788 7494560

[74] Remuzzi A Gagliardini E Sangalli F Bonomelli M Piccinelli M Benigni A ACE inhibition reduces glomerulosclerosis and regenerates glomerular tissue in a model of progressive renal disease. *Kidney Int.* (2006) 69:1124–30. 10.1038/sj.ki.5000060 16395266

[75] Bangalore S Fakheri R Wandel S Toklu B Wandel J Messerli FH. Renin angiotensin system inhibitors for patients with stable coronary artery disease without heart failure: systematic review and meta-analysis of randomized trials. *BMJ.* (2017) 356:j4. 10.1136/bmj.j4 28104622 PMC5244819

[76] Agarwal R Kolkhof P Bakris G Bauersachs J Haller H Wada T Steroidal and non-steroidal mineralocorticoid receptor antagonists in cardiorenal medicine. *Eur Heart J.* (2021) 42:152–61. 10.1093/eurheartj/ehaa736 33099609 PMC7813624

[77] Colafella KMM Hilliard LM Denton KM. Epochs in the depressor/pressor balance of the renin-angiotensin system. *Clin Sci.* (2016) 130:761–71. 10.1042/CS20150939 27128801

[78] Santos RA Campagnole-Santos MJ Andrade SP. Angiotensin-(1-7): an update. *Regul Pept.* (2000) 91:45–62. 10.1016/s0167-0115(00)00138-5 10967201

[79] Clarke NE Turner AJ. Angiotensin-converting enzyme 2: the first decade. *Int J Hypertens.* (2012) 2012:307315. 10.1155/2012/307315 22121476 PMC3216391

[80] Tipnis SR Hooper NM Hyde R Karran E Christie G Turner AJ. A human homolog of angiotensin-converting enzyme. Cloning and functional expression as a captopril-insensitive carboxypeptidase. *J Biol Chem.* (2000) 275:33238–43. 10.1074/jbc.M002615200 10924499

[81] Vickers C Hales P Kaushik V Dick L Gavin J Tang J Hydrolysis of biological peptides by human angiotensin-converting enzyme-related carboxypeptidase. *J Biol Chem.* (2002) 277:14838–43. 10.1074/jbc.M200581200 11815627

[82] Crackower MA Sarao R Oudit GY Yagil C Kozieradzki I Scanga SE Angiotensin-converting enzyme 2 is an essential regulator of heart function. *Nature.* (2002) 417:822–8. 10.1038/nature00786 12075344

[83] Hamming I Cooper ME Haagmans BL Hooper NM Korstanje R Osterhaus ADME The emerging role of ACE2 in physiology and disease. *J Pathol.* (2007) 212:1–11. 10.1002/path.2162 17464936 PMC7167724

[84] Santos RAS Ferreira AJ Simões E Silva AC. Recent advances in the angiotensin-converting enzyme 2-angiotensin(1-7)-Mas axis. *Exp Physiol.* (2008) 93:519–27. 10.1113/expphysiol.2008.042002 18310257

[85] Schrier RW Abraham WT. Hormones and hemodynamics in heart failure. *N Engl J Med.* (1999) 341:577–85. 10.1056/NEJM199908193410806 10451464

[86] Myers BD Deen WM Brenner BM. Effects of norepinephrine and angiotensin II on the determinants of glomerular ultrafiltration and proximal tubule fluid reabsorption in the rat. *Circ Res.* (1975) 37:101–10. 10.1161/01.res.37.1.101 1149180

[87] Schoolwerth AC Sica DA Ballermann BJ Wilcox CS. Renal considerations in angiotensin converting enzyme inhibitor therapy: a statement for healthcare professionals from the council on the kidney in cardiovascular disease and the council for high blood pressure research of the American Heart Association. *Circulation.* (2001) 104:1985–91. 10.1161/hc4101.096153 11602506

[88] Sica DA. Edema mechanisms in the patient with heart failure and treatment options. *Heart Fail Clin.* (2008) 4:511–8. 10.1016/j.hfc.2008.04.002 18760761

[89] Beldhuis IE Streng KW Ter Maaten JM Voors AA van der Meer P Rossignol P Renin-angiotensin system inhibition, worsening renal function, and outcome in heart failure patients with reduced and preserved ejection fraction: a meta-analysis of published study data. *Circ Heart Fail.* (2017) 10:e003588. 10.1161/CIRCHEARTFAILURE.116.003588 28209765

[90] Abassi Z Assady S Khoury EE Heyman SN. Letter to the editor: angiotensin-converting enzyme 2: an ally or a Trojan horse? implications to SARS-CoV-2-related cardiovascular complications. *Am J Physiol Heart Circ Physiol.* (2020) 318:H1080–3. 10.1152/ajpheart.00215.2020 32223552 PMC7191629

[91] Lee VCH Lloyd EN Dearden HC Wong K. A systematic review to investigate whether Angiotensin-(1-7) is a promising therapeutic target in human heart failure. *Int J Pept.* (2013) 2013:260346. 10.1155/2013/260346 24454410 PMC3876703

[92] Tripathi R Sullivan RD Fan T-HM Mehta RM Gladysheva IP Reed GL. A Low-sodium diet boosts Ang (1-7) production and NO-cGMP bioavailability to reduce edema and enhance survival in experimental heart failure. *Int J Mol Sci.* (2021) 22:4035. 10.3390/ijms22084035 33919841 PMC8070795

[93] DiBona GF Kopp UC. Neural control of renal function. *Physiol Rev.* (1997) 77:75–197. 10.1152/physrev.1997.77.1.75 9016301

[94] Denton KM Luff SE Shweta A Anderson WP. Differential neural control of glomerular ultrafiltration. *Clin Exp Pharmacol Physiol.* (2004) 31:380–6. 10.1111/j.1440-1681.2004.04002.x 15191417

[95] Kaye D Esler M. Sympathetic neuronal regulation of the heart in aging and heart failure. *Cardiovasc Res.* (2005) 66:256–64. 10.1016/j.cardiores.2005.02.012 15820194

[96] Watson AMD Hood SG May CN. Mechanisms of sympathetic activation in heart failure. *Clin Exp Pharmacol Physiol.* (2006) 33:1269–74. 10.1111/j.1440-1681.2006.04523.x 17184514

[97] DiBona GF. Peripheral and central interactions between the renin-angiotensin system and the renal sympathetic nerves in control of renal function. *Ann N Y Acad Sci.* (2001) 940:395–406. 10.1111/j.1749-6632.2001.tb03693.x 11458695

[98] Kon V Yared A Ichikawa I. Role of renal sympathetic nerves in mediating hypoperfusion of renal cortical microcirculation in experimental congestive heart failure and acute extracellular fluid volume depletion. *J Clin Invest.* (1985) 76:1913–20. 10.1172/JCI112187 4056058 PMC424240

[99] Dupont AG. Effects of carvedilol on renal function. *Eur J Clin Pharmacol.* (1990) 38(Suppl. 2):S96–100. 10.1007/BF01409473 1974513

[100] Heitmann M Davidsen U Stokholm KH Rasmussen K Burchardt H Petersen EB. Renal and cardiac function during alpha1-beta-blockade in congestive heart failure. *Scand J Clin Lab Invest.* (2002) 62:97–104. 10.1080/003655102753611717 12004934

[101] Chen HH Schrier RW. Pathophysiology of volume overload in acute heart failure syndromes. *Am J Med.* (2006) 119:S11–6. 10.1016/j.amjmed.2006.09.012 17113395

[102] Szatalowicz VL Arnold PE Chaimovitz C Bichet D Berl T Schrier RW. Radioimmunoassay of plasma arginine vasopressin in hyponatremic patients with congestive heart failure. *N Engl J Med.* (1981) 305:263–6. 10.1056/NEJM198107303050506 7242616

[103] Schrier RW. Role of diminished renal function in cardiovascular mortality: marker or pathogenetic factor? *J Am Coll Cardiol.* (2006) 47:1–8. 10.1016/j.jacc.2005.07.067 16386657

[104] Noda Y Sasaki S. Updates and perspectives on aquaporin-2 and water balance disorders. *Int J Mol Sci.* (2021) 22:12950. 10.3390/ijms222312950 34884753 PMC8657825

[105] Goldsmith SR. Arginine vasopressin antagonism in heart failure: current status and possible new directions. *J Cardiol.* (2019) 74:49–52. 10.1016/j.jjcc.2019.03.001 30904236

[106] Kohan DE. The renal medullary endothelin system in control of sodium and water excretion and systemic blood pressure. *Curr Opin Nephrol Hypertens.* (2006) 15:34–40. 10.1097/01.mnh.0000186852.15889.1a 16340664

[107] Masaki T. Possible role of endothelin in endothelial regulation of vascular tone. *Annu Rev Pharmacol Toxicol.* (1995) 35:235–55. 10.1146/annurev.pa.35.040195.001315 7598493

[108] Eroglu E Kocyigit I Lindholm B. The endothelin system as target for therapeutic interventions in cardiovascular and renal disease. *Clin Chim Acta.* (2020) 506:92–106. 10.1016/j.cca.2020.03.008 32151622

[109] Brunner F Brás-Silva C Cerdeira AS Leite-Moreira AF. Cardiovascular endothelins: essential regulators of cardiovascular homeostasis. *Pharmacol Ther.* (2006) 111:508–31. 10.1016/j.pharmthera.2005.11.001 16457892

[110] Kedzierski RM Yanagisawa M. Endothelin system: the double-edged sword in health and disease. *Annu Rev Pharmacol Toxicol.* (2001) 41:851–76. 10.1146/annurev.pharmtox.41.1.851 11264479

[111] Pugliese NR Fabiani I Conte L Nesti L Masi S Natali A Persistent congestion, renal dysfunction and inflammatory cytokines in acute heart failure: a prognosis study. *J Cardiovasc Med.* (2020) 21:494–502. 10.2459/JCM.0000000000000974 32487865

[112] McMurray JJ Ray SG Abdullah I Dargie HJ Morton JJ. Plasma endothelin in chronic heart failure. *Circulation.* (1992) 85:1374–9. 10.1161/01.cir.85.4.1374 1532540

[113] Gurbanov K Rubinstein I Hoffman A Abassi Z Better OS Winaver J. Bosentan improves renal regional blood flow in rats with experimental congestive heart failure. *Eur J Pharmacol.* (1996) 310:193–6. 10.1016/0014-2999(96)00494-3 8884216

[114] Qiu C Ding SS Hess P Clozel JP Clozel M. Endothelin mediates the altered renal hemodynamics associated with experimental congestive heart failure. *J Cardiovasc Pharmacol.* (2001) 38:317–24. 10.1097/00005344-200108000-00017 11483881

[115] Bauersachs J Braun C Fraccarollo D Widder J Ertl G Schilling L Improvement of renal dysfunction in rats with chronic heart failure after myocardial infarction by treatment with the endothelin A receptor antagonist, LU 135252. *J Hypertens.* (2000) 18:1507–14. 10.1097/00004872-200018100-00020 11057440

[116] Borgeson DD Grantham JA Williamson EE Luchner A Redfield MM Opgenorth TJ Chronic oral endothelin type A receptor antagonism in experimental heart failure. *Hypertension.* (1998) 31:766–70. 10.1161/01.hyp.31.3.766 9495259

[117] Ding S-S Qiu C Hess P Xi J-F Clozel J-P Clozel M. Chronic endothelin receptor blockade prevents renal vasoconstriction and sodium retention in rats with chronic heart failure. *Cardiovasc Res.* (2002) 53:963–70. 10.1016/s0008-6363(01)00558-2 11922906

[118] Packer M McMurray JJV Krum H Kiowski W Massie BM Caspi A Long-term effect of endothelin receptor antagonism with bosentan on the morbidity and mortality of patients with severe chronic heart failure: primary results of the ENABLE trials. *JACC Heart Fail.* (2017) 5:317–26. 10.1016/j.jchf.2017.02.021 28449795

[119] Goetze JP Bruneau BG Ramos HR Ogawa T de Bold MK de Bold AJ. Cardiac natriuretic peptides. *Nat Rev Cardiol.* (2020) 17:698–717. 10.1038/s41569-020-0381-0 32444692

[120] Moyes AJ Hobbs AJ. C-type natriuretic peptide: a multifaceted paracrine regulator in the heart and vasculature. *Int J Mol Sci.* (2019) 20:2281. 10.3390/ijms20092281 31072047 PMC6539462

[121] Nakagawa Y Nishikimi T Kuwahara K. Atrial and brain natriuretic peptides: hormones secreted from the heart. *Peptides.* (2019) 111:18–25. 10.1016/j.peptides.2018.05.012 29859763

[122] Zeller R Bloch KD Williams BS Arceci RJ Seidman CE. Localized expression of the atrial natriuretic factor gene during cardiac embryogenesis. *Genes Dev.* (1987) 1:693–8. 10.1101/gad.1.7.693 2962900

[123] Semenov AG Tamm NN Seferian KR Postnikov AB Karpova NS Serebryanaya DV Processing of pro-B-type natriuretic peptide: furin and corin as candidate convertases. *Clin Chem.* (2010) 56:1166–76. 10.1373/clinchem.2010.143883 20489134

[124] Wu F Yan W Pan J Morser J Wu Q. Processing of pro-atrial natriuretic peptide by corin in cardiac myocytes. *J Biol Chem.* (2002) 277:16900–5. 10.1074/jbc.M201503200 11884416

[125] Yan W Wu F Morser J Wu Q. Corin, a transmembrane cardiac serine protease, acts as a pro-atrial natriuretic peptide-converting enzyme. *Proc Natl Acad Sci U.S.A.* (2000) 97:8525–9. 10.1073/pnas.150149097 10880574 PMC26981

[126] Gladysheva IP Robinson BR Houng AK Kováts T King SM. Corin is co-expressed with pro-ANP and localized on the cardiomyocyte surface in both zymogen and catalytically active forms. *J Mol Cell Cardiol.* (2008) 44:131–42. 10.1016/j.yjmcc.2007.10.002 17996891

[127] Bensimon M Chang AI de Bold MLK Ponce A Carreras D De Bold AJ. Participation of G proteins in natriuretic peptide hormone secretion from heart atria. *Endocrinology.* (2004) 145:5313–21. 10.1210/en.2004-0698 15308619

[128] Ogawa T Vatta M Bruneau BG de Bold AJ. Characterization of natriuretic peptide production by adult heart atria. *Am J Physiol.* (1999) 276:H1977–86. 10.1152/ajpheart.1999.276.6.H1977 10362678

[129] Ma KK Ogawa T de Bold AJ. Selective upregulation of cardiac brain natriuretic peptide at the transcriptional and translational levels by pro-inflammatory cytokines and by conditioned medium derived from mixed lymphocyte reactions *via* p38 MAP kinase. *J Mol Cell Cardiol.* (2004) 36:505–13. 10.1016/j.yjmcc.2004.01.001 15081310

[130] Mangat H de Bold AJ. Stretch-induced atrial natriuretic factor release utilizes a rapidly depleting pool of newly synthesized hormone. *Endocrinology.* (1993) 133:1398–403. 10.1210/endo.133.3.8365374 8365374

[131] Burnett JCJ Kao PC Hu DC Heser DW Heublein D Granger JP Atrial natriuretic peptide elevation in congestive heart failure in the human. *Science.* (1986) 231:1145–7. 10.1126/science.2935937 2935937

[132] Yandle TG Richards AM Gilbert A Fisher S Holmes S Espiner EA. Assay of brain natriuretic peptide (BNP) in human plasma: evidence for high molecular weight BNP as a major plasma component in heart failure. *J Clin Endocrinol Metab.* (1993) 76:832–8. 10.1210/jcem.76.4.8473392 8473392

[133] Huntley BK Sandberg SM Heublein DM Sangaralingham SJ Burnett JCJ Ichiki T. Pro-B-type natriuretic peptide-1-108 processing and degradation in human heart failure. *Circ Heart Fail.* (2015) 8:89–97. 10.1161/CIRCHEARTFAILURE.114.001174 25339504 PMC4303547

[134] McMurray JJV Adamopoulos S Anker SD Auricchio A Böhm M Dickstein K ESC guidelines for the diagnosis and treatment of acute and chronic heart failure 2012: the task force for the diagnosis and treatment of acute and chronic heart failure 2012 of the European Society of Cardiology. Developed in collaboration with the Heart Failure Association (HFA) of the ESC. *Eur Heart J.* (2012) 33:1787–847. 10.1093/eurheartj/ehs104 22611136

[135] Yancy CW Jessup M Bozkurt B Butler J Casey DEJ Drazner MH 2013 ACCF/AHA guideline for the management of heart failure: a report of the American College of Cardiology Foundation/American Heart Association task force on practice guidelines. *J Am Coll Cardiol.* (2013) 62:e147–239. 10.1016/j.jacc.2013.05.019 23747642

[136] Braunwald E. Biomarkers in heart failure. *N Engl J Med.* (2008) 358:2148–59. 10.1056/NEJMra0800239 18480207

[137] Lee KK Doudesis D Anwar M Astengo F Chenevier-Gobeaux C Claessens Y-E Development and validation of a decision support tool for the diagnosis of acute heart failure: systematic review, meta-analysis, and modelling study. *BMJ.* (2022) 377:e068424. 10.1136/bmj-2021-068424 35697365 PMC9189738

[138] Charloux A Piquard F Doutreleau S Brandenberger G Geny B. Mechanisms of renal hyporesponsiveness to ANP in heart failure. *Eur J Clin Invest.* (2003) 33:769–78. 10.1046/j.1365-2362.2003.01222.x 12925036

[139] Egom EE Feridooni T Hotchkiss A Kruzliak P Pasumarthi KBS. Mechanisms of renal hyporesponsiveness to BNP in heart failure. *Can J Physiol Pharmacol.* (2015) 93:399–403. 10.1139/cjpp-2014-0356 25881664

[140] Damman K Navis G Smilde TDJ Voors AA van der Bij W van Veldhuisen DJ Decreased cardiac output, venous congestion and the association with renal impairment in patients with cardiac dysfunction. *Eur J Heart Fail.* (2007) 9:872–8. 10.1016/j.ejheart.2007.05.010 17586090

[141] Nakagawa Y Nishikimi T Kuwahara K Fujishima A Oka S Tsutamoto T MiR30-GALNT1/2 axis-mediated glycosylation contributes to the increased secretion of inactive human prohormone for brain natriuretic peptide (proBNP) from failing hearts. *J Am Heart Assoc.* (2017) 6:e003601. 10.1161/JAHA.116.003601 28188250 PMC5523735

[142] Peng J Jiang J Wang W Qi X Sun X-L Wu Q. Glycosylation and processing of pro-B-type natriuretic peptide in cardiomyocytes. *Biochem Biophys Res Commun.* (2011) 411:593–8. 10.1016/j.bbrc.2011.06.192 21763278 PMC3152652

[143] Tonne JM Campbell JM Cataliotti A Ohmine S Thatava T Sakuma T Secretion of glycosylated pro-B-type natriuretic peptide from normal cardiomyocytes. *Clin Chem.* (2011) 57:864–73. 10.1373/clinchem.2010.157438 21482747 PMC3634583

[144] Schellenberger U O’Rear J Guzzetta A Jue RA Protter AA Pollitt NS. The precursor to B-type natriuretic peptide is an O-linked glycoprotein. *Arch Biochem Biophys.* (2006) 451:160–6. 10.1016/j.abb.2006.03.028 16750161

[145] Semenov AG Postnikov AB Tamm NN Seferian KR Karpova NS Bloshchitsyna MN Processing of pro-brain natriuretic peptide is suppressed by O-glycosylation in the region close to the cleavage site. *Clin Chem.* (2009) 55:489–98. 10.1373/clinchem.2008.113373 19168558

[146] Hansen LH Madsen TD Goth CK Clausen H Chen Y Dzhoyashvili N Discovery of O-glycans on atrial natriuretic peptide (ANP) that affect both its proteolytic degradation and potency at its cognate receptor. *J Biol Chem.* (2019) 294:12567–78. 10.1074/jbc.RA119.008102 31186350 PMC6709625

[147] Vodovar N Séronde M-F Laribi S Gayat E Lassus J Boukef R Post-translational modifications enhance NT-proBNP and BNP production in acute decompensated heart failure. *Eur Heart J.* (2014) 35:3434–41. 10.1093/eurheartj/ehu314 25157115

[148] Brandt I Lambeir A-M Ketelslegers J-M Vanderheyden M Scharpé S De Meester I. Dipeptidyl-peptidase IV converts intact B-type natriuretic peptide into its des-SerPro form. *Clin Chem.* (2006) 52:82–7. 10.1373/clinchem.2005.057638 16254193

[149] Boerrigter G Costello-Boerrigter LC Harty GJ Lapp H Burnett JCJ. Des-serine-proline brain natriuretic peptide 3-32 in cardiorenal regulation. *Am J Physiol Regul Integr Comp Physiol.* (2007) 292:R897–901. 10.1152/ajpregu.00569.2006 17068158

[150] Pankow K Wang Y Gembardt F Krause E Sun X Krause G Successive action of meprin A and neprilysin catabolizes B-type natriuretic peptide. *Circ Res.* (2007) 101:875–82. 10.1161/CIRCRESAHA.107.153585 17823376

[151] Ralat LA Guo Q Ren M Funke T Dickey DM Potter LR Insulin-degrading enzyme modulates the natriuretic peptide-mediated signaling response. *J Biol Chem.* (2011) 286:4670–9. 10.1074/jbc.M110.173252 21098034 PMC3039328

[152] Toll L Brandt SR Olsen CM Judd AK Almquist RG. Isolation and characterization of a new atrial peptide-degrading enzyme from bovine kidney. *Biochem Biophys Res Commun.* (1991) 175:886–93. 10.1016/0006-291x(91)91648-v 1850994

[153] Müller D Baumeister H Buck F Richter D. Atrial natriuretic peptide (ANP) is a high-affinity substrate for rat insulin-degrading enzyme. *Eur J Biochem.* (1991) 202:285–92. 10.1111/j.1432-1033.1991.tb16374.x 1836994

[154] Miller WL Phelps MA Wood CM Schellenberger U Van Le A Perichon R Comparison of mass spectrometry and clinical assay measurements of circulating fragments of B-type natriuretic peptide in patients with chronic heart failure. *Circ Heart Fail.* (2011) 4:355–60. 10.1161/CIRCHEARTFAILURE.110.960260 21292992

[155] Clerico A Vittorini S Passino C. Circulating forms of the b-type natriuretic peptide prohormone: pathophysiologic and clinical considerations. *Adv Clin Chem.* (2012) 58:31–44. 10.1016/b978-0-12-394383-5.00008-4 22950341

[156] dos Santos L Salles TA Arruda-Junior DF Campos LCG Pereira AC Barreto ALT Circulating dipeptidyl peptidase IV activity correlates with cardiac dysfunction in human and experimental heart failure. *Circ Heart Fail.* (2013) 6:1029–38. 10.1161/CIRCHEARTFAILURE.112.000057 23894014

[157] Bayés-Genís A Barallat J Galán A de Antonio M Domingo M Zamora E Soluble neprilysin is predictive of cardiovascular death and heart failure hospitalization in heart failure patients. *J Am Coll Cardiol.* (2015) 65:657–65. 10.1016/j.jacc.2014.11.048 25677426

[158] Knecht M Pagel I Langenickel T Philipp S Scheuermann-Freestone M Willnow T Increased expression of renal neutral endopeptidase in severe heart failure. *Life Sci.* (2002) 71:2701–12. 10.1016/s0024-3205(02)01990-2 12383878

[159] Solomon SD McMurray JJV Anand IS Ge J Lam CSP Maggioni AP Angiotensin-neprilysin inhibition in heart failure with preserved ejection fraction. *N Engl J Med.* (2019) 381:1609–20. 10.1056/NEJMoa1908655 31475794

[160] McMurray JJV Packer M Desai AS Gong J Lefkowitz MP Rizkala AR Angiotensin-neprilysin inhibition versus enalapril in heart failure. *N Engl J Med.* (2014) 371:993–1004. 10.1056/NEJMoa1409077 25176015

[161] Velazquez EJ Morrow DA DeVore AD Duffy CI Ambrosy AP McCague K Angiotensin-neprilysin inhibition in acute decompensated heart failure. *N Engl J Med.* (2019) 380:539–48. 10.1056/NEJMoa1812851 30415601

[162] Kukkonen P Vuolteenaho O Ruskoaho H. Basal and volume expansion-stimulated plasma atrial natriuretic peptide concentrations and hemodynamics in conscious rats: effects of SCH 39.370, an endopeptidase inhibitor, and C-ANF-(4-23), a clearance receptor ligand. *Endocrinology.* (1992) 130:755–65. 10.1210/endo.130.2.1531129 1531129

[163] Wang DJ Dowling TC Meadows D Ayala T Marshall J Minshall S Nesiritide does not improve renal function in patients with chronic heart failure and worsening serum creatinine. *Circulation.* (2004) 110:1620–5. 10.1161/01.CIR.0000141829.04031.25 15337695

[164] Ichiki T Huntley BK Heublein DM Sandberg SM McKie PM Martin FL Corin is present in the normal human heart, kidney, and blood, with pro-B-type natriuretic peptide processing in the circulation. *Clin Chem.* (2011) 57:40–7. 10.1373/clinchem.2010.153908 21075870

[165] Yan W Sheng N Seto M Morser J Wu Q. Corin, a mosaic transmembrane serine protease encoded by a novel cDNA from human heart. *J Biol Chem.* (1999) 274:14926–35. 10.1074/jbc.274.21.14926 10329693

[166] Chan JCY Knudson O Wu F Morser J Dole WP Wu Q. Hypertension in mice lacking the proatrial natriuretic peptide convertase corin. *Proc Natl Acad Sci U.S.A.* (2005) 102:785–90. 10.1073/pnas.0407234102 15637153 PMC545541

[167] Buckley CL Stokes AJ. Corin-deficient W-sh mice poorly tolerate increased cardiac afterload. *Regul Pept.* (2011) 172:44–50. 10.1016/j.regpep.2011.08.006 21903139 PMC3196309

[168] Gladysheva IP Wang D McNamee RA Houng AK Mohamad AA Fan TM Corin overexpression improves cardiac function, heart failure, and survival in mice with dilated cardiomyopathy. *Hypertension.* (2013) 61:327–32. 10.1161/HYPERTENSIONAHA.112.193631 23232642 PMC3728819

[169] Rame JE Drazner MH Post W Peshock R Lima J Cooper RS Corin I555(P568) allele is associated with enhanced cardiac hypertrophic response to increased systemic afterload. *Hypertension.* (2007) 49:857–64. 10.1161/01.HYP.0000258566.95867.9e 17296875

[170] Knappe S Wu F Madlansacay MR Wu Q. Identification of domain structures in the propeptide of corin essential for the processing of proatrial natriuretic peptide. *J Biol Chem.* (2004) 279:34464–71. 10.1074/jbc.M405041200 15192093

[171] Dries DL Victor RG Rame JE Cooper RS Wu X Zhu X Corin gene minor allele defined by 2 missense mutations is common in blacks and associated with high blood pressure and hypertension. *Circulation.* (2005) 112:2403–10. 10.1161/CIRCULATIONAHA.105.568881 16216958

[172] Wang W Liao X Fukuda K Knappe S Wu F Dries DL Corin variant associated with hypertension and cardiac hypertrophy exhibits impaired zymogen activation and natriuretic peptide processing activity. *Circ Res.* (2008) 103:502–8. 10.1161/CIRCRESAHA.108.177352 18669922 PMC2652846

[173] Wang W Cui Y Shen J Jiang J Chen S Peng J Salt-sensitive hypertension and cardiac hypertrophy in transgenic mice expressing a corin variant identified in blacks. *Hypertension.* (2012) 60:1352–8. 10.1161/HYPERTENSIONAHA.112.201244 22987923 PMC3475733

[174] Tran KL Lu X Lei M Feng Q Wu Q. Upregulation of corin gene expression in hypertrophic cardiomyocytes and failing myocardium. *Am J Physiol Heart Circ Physiol.* (2004) 287:H1625–31. 10.1152/ajpheart.00298.2004 15191894

[175] Calderone A Bel-Hadj S Drapeau J El-Helou V Gosselin H Clement R Scar myofibroblasts of the infarcted rat heart express natriuretic peptides. *J Cell Physiol.* (2006) 207:165–73. 10.1002/jcp.20548 16270351

[176] Jiang W Cai D-Y Pan C-S Qi Y-F Jiang H-F Geng B Changes in production and metabolism of brain natriuretic peptide in rats with myocardial necrosis. *Eur J Pharmacol.* (2005) 507:153–62. 10.1016/j.ejphar.2004.11.023 15659305

[177] Chen S Sen S Young D Wang W Moravec CS Wu Q. Protease corin expression and activity in failing hearts. *Am J Physiol Heart Circ Physiol.* (2010) 299:H1687–92. 10.1152/ajpheart.00399.2010 20802129 PMC2993205

[178] Langenickel TH Pagel I Buttgereit J Tenner K Lindner M Dietz R Rat corin gene: molecular cloning and reduced expression in experimental heart failure. *Am J Physiol Heart Circ Physiol.* (2004) 287:H1516–21. 10.1152/ajpheart.00947.2003 15155264

[179] Ichiki T Boerrigter G Huntley BK Sangaralingham SJ McKie PM Harty GJ Differential expression of the pro-natriuretic peptide convertases corin and furin in experimental heart failure and atrial fibrosis. *Am J Physiol Regul Integr Comp Physiol.* (2013) 304:R102–9. 10.1152/ajpregu.00233.2012 23152112 PMC3543660

[180] Tripathi R Wang D Sullivan R Fan T-HM Gladysheva IP Reed GL. Depressed corin levels indicate early systolic dysfunction before increases of atrial natriuretic peptide/B-type natriuretic peptide and heart failure development. *Hypertension.* (2016) 67:362–7. 10.1161/HYPERTENSIONAHA.115.06300 26667411 PMC4790413

[181] Ngo DTM Horowitz JD Sverdlov AL. Heart failure: a corin-deficient state? *Hypertension.* (2013) 61:284–5. 10.1161/HYPERTENSIONAHA.112.196253 23232647

[182] Tripathi R Sullivan RD Fan T-HM Houng AK Mehta RM Reed GL Cardiac-specific overexpression of catalytically inactive corin reduces edema, contractile dysfunction, and death in mice with dilated cardiomyopathy. *Int J Mol Sci.* (2019) 21:203. 10.3390/ijms21010203 31892216 PMC6981738

[183] Peleg A Ghanim D Vered S Hasin Y. Serum corin is reduced and predicts adverse outcome in non-ST-elevation acute coronary syndrome. *Eur Heart J Acute Cardiovasc Care.* (2013) 2:159–65. 10.1177/2048872613483588 24222826 PMC3821806

[184] Dong N Chen S Yang J He L Liu P Zheng D Plasma soluble corin in patients with heart failure. *Circ Heart Fail.* (2010) 3:207–11. 10.1161/CIRCHEARTFAILURE.109.903849 20061521 PMC2879139

[185] Zhou X Chen J-C Liu Y Yang H Du K Kong Y Plasma corin as a predictor of cardiovascular events in patients with chronic heart failure. *JACC Heart Fail.* (2016) 4:664–9. 10.1016/j.jchf.2016.03.006 27179834

[186] Yu Z Lu X Xu W Jin M Tao Y Zhou X. Serum corin is associated with the risk of chronic heart failure. *Oncotarget.* (2017) 8:100353–7. 10.18632/oncotarget.22227 29245983 PMC5725025

[187] Gommans DHF Revuelta-Lopez E Lupon J Cserkóová A Domingo M Vart P Soluble neprilysin and corin concentrations in relation to clinical outcome in chronic heart failure. *JACC Heart Fail.* (2021) 9:85–95. 10.1016/j.jchf.2020.08.015 33189629

[188] Zhou X Chen J Zhang Q Shao J Du K Xu X Prognostic value of plasma soluble corin in patients with acute myocardial infarction. *J Am Coll Cardiol.* (2016) 67:2008–14. 10.1016/j.jacc.2016.02.035 27126527

[189] Feistritzer H-J Metzler B. Corin as novel biomarker for myocardial infarction. *Ann Transl Med.* (2016) 4:405. 10.21037/atm.2016.08.17 27867957 PMC5107398

[190] Chen F Xia Y Liu Y Zhang Y Song W Zhong Y Increased plasma corin levels in patients with atrial fibrillation. *Clin Chim Acta.* (2015) 447:79–85. 10.1016/j.cca.2015.05.017 26048191

[191] Ibebuogu UN Gladysheva IP Houng AK Reed GL. Decompensated heart failure is associated with reduced corin levels and decreased cleavage of pro-atrial natriuretic peptide. *Circ Heart Fail.* (2011) 4:114–20. 10.1161/CIRCHEARTFAILURE.109.895581 21216831 PMC3840730

[192] Wang D Gladysheva IP Sullivan RD Fan T-HM Mehta RM Tripathi R Increases in plasma corin levels following experimental myocardial infarction reflect the severity of ischemic injury. *PLoS One.* (2018) 13:e0202571. 10.1371/journal.pone.0202571 30192780 PMC6128455

[193] Dries DL. Process matters: emerging concepts underlying impaired natriuretic peptide system function in heart failure. *Circ Heart Fail.* (2011) 4:107–10. 10.1161/CIRCHEARTFAILURE.111.960948 21406676

[194] Zaidi SS Ward RD Ramanathan K Yu X Gladysheva IP Reed GL. Possible enzymatic downregulation of the natriuretic peptide system in patients with reduced systolic function and heart failure: a pilot study. *Biomed Res Int.* (2018) 2018:7279036. 10.1155/2018/7279036 30148170 PMC6083548

[195] Verstreken S Delrue L Goethals M Bartunek J Vanderheyden M. Natriuretic peptide processing in patients with and without left ventricular dysfunction. *Int Heart J.* (2019) 60:115–20. 10.1536/ihj.18-012 30518715

[196] Dong N Chen S Wang W Zhou Y Wu Q. Corin in clinical laboratory diagnostics. *Clin Chim Acta.* (2012) 413:378–83. 10.1016/j.cca.2011.10.032 22093942 PMC3246062

[197] Chen S Cao P Dong N Peng J Zhang C Wang H PCSK6-mediated corin activation is essential for normal blood pressure. *Nat Med.* (2015) 21:1048–53. 10.1038/nm.3920 26259032 PMC4710517

[198] Khoury EE Fokra A Kinaneh S Knaney Y Aronson D Abassi Z. Distribution of cardiac and renal corin and proprotein convertase subtilisin/kexin-6 in the experimental model of cardio-renal syndrome of various severities. *Front Physiol.* (2021) 12:673497. 10.3389/fphys.2021.673497 34733169 PMC8558519

[199] Ichiki T Huntley BK Burnett JCJ. BNP molecular forms and processing by the cardiac serine protease corin. *Adv Clin Chem.* (2013) 61:1–31. 10.1016/b978-0-12-407680-8.00001-4 24015598 PMC4522930

[200] Giuliani I Rieunier F Larue C Delagneau J-F Granier C Pau B Assay for measurement of intact B-type natriuretic peptide prohormone in blood. *Clin Chem.* (2006) 52:1054–61. 10.1373/clinchem.2005.061770 16574763

[201] Lam CSP Burnett JCJ Costello-Boerrigter L Rodeheffer RJ Redfield MM. Alternate circulating pro-B-type natriuretic peptide and B-type natriuretic peptide forms in the general population. *J Am Coll Cardiol.* (2007) 49:1193–202. 10.1016/j.jacc.2006.12.024 17367664

[202] Dickey DM Potter LR. ProBNP(1-108) is resistant to degradation and activates guanylyl cyclase-A with reduced potency. *Clin Chem.* (2011) 57:1272–8. 10.1373/clinchem.2011.169151 21768217 PMC4855511

[203] Hawkridge AM Heublein DM Bergen HR III Cataliotti A Burnett JCJ Muddiman DC. Quantitative mass spectral evidence for the absence of circulating brain natriuretic peptide (BNP-32) in severe human heart failure. *Proc Natl Acad Sci U.S.A.* (2005) 102:17442–7. 10.1073/pnas.0508782102 16293687 PMC1297688

[204] Seferian KR Tamm NN Semenov AG Mukharyamova KS Tolstaya AA Koshkina EV The brain natriuretic peptide (BNP) precursor is the major immunoreactive form of BNP in patients with heart failure. *Clin Chem.* (2007) 53:866–73. 10.1373/clinchem.2006.076141 17384012

[205] Winton FR. The influence of venous pressure on the isolated mammalian kidney. *J Physiol.* (1931) 72:49–61. 10.1113/jphysiol.1931.sp002761 16994199 PMC1403105

[206] Damman K van Deursen VM Navis G Voors AA van Veldhuisen DJ Hillege HL. Increased central venous pressure is associated with impaired renal function and mortality in a broad spectrum of patients with cardiovascular disease. *J Am Coll Cardiol.* (2009) 53:582–8. 10.1016/j.jacc.2008.08.080 19215832

[207] Kitani T Kidokoro K Nakata T Kirita Y Nakamura I Nakai K Kidney vascular congestion exacerbates acute kidney injury in mice. *Kidney Int.* (2022) 101:551–62. 10.1016/j.kint.2021.11.015 34843756

[208] Fiksen-Olsen MJ Strick DM Hawley H Romero JC. Renal effects of angiotensin II inhibition during increases in renal venous pressure. *Hypertension.* (1992) 19:II137–41. 10.1161/01.hyp.19.2_suppl.ii137 1735568

[209] Burnett JCJ Knox FG. Renal interstitial pressure and sodium excretion during renal vein constriction. *Am J Physiol.* (1980) 238:F279–82. 10.1152/ajprenal.1980.238.4.F279 7377299

[210] Firth JD Raine AE Ledingham JG. Raised venous pressure: a direct cause of renal sodium retention in oedema? *Lancet.* (1988) 1:1033–5. 10.1016/s0140-6736(88)91851-x 2896877

[211] Afsar B Ortiz A Covic A Solak Y Goldsmith D Kanbay M. Focus on renal congestion in heart failure. *Clin Kidney J.* (2016) 9:39–47. 10.1093/ckj/sfv124 26798459 PMC4720202

[212] Boorsma EM Ter Maaten JM Voors AA van Veldhuisen DJ. Renal compression in heart failure: the renal tamponade hypothesis. *JACC Heart Fail.* (2022) 10:175–83. 10.1016/j.jchf.2021.12.005 35241245

[213] Kastner PR Hall JE Guyton AC. Renal hemodynamic responses to increased renal venous pressure: role of angiotensin II. *Am J Physiol.* (1982) 243:F260–4. 10.1152/ajprenal.1982.243.3.F260 7051857

[214] Doty JM Saggi BH Sugerman HJ Blocher CR Pin R Fakhry I Effect of increased renal venous pressure on renal function. *J Trauma.* (1999) 47:1000–3. 10.1097/00005373-199912000-00002 10608524

[215] Bishara B Abu-Saleh N Awad H Ghrayeb N Goltsman I Aronson D Phosphodiesterase 5 inhibition protects against increased intra-abdominal pressure-induced renal dysfunction in experimental congestive heart failure. *Eur J Heart Fail.* (2012) 14:1104–11. 10.1093/eurjhf/hfs102 22740510

[216] Husain-Syed F Gröne H-J Assmus B Bauer P Gall H Seeger W Congestive nephropathy: a neglected entity? Proposal for diagnostic criteria and future perspectives. *ESC Hear Fail.* (2021) 8:183–203. 10.1002/ehf2.13118 33258308 PMC7835563

[217] Webb DJ Vachiery J-L Hwang L-J Maurey JO. Sildenafil improves renal function in patients with pulmonary arterial hypertension. *Br J Clin Pharmacol.* (2015) 80:235–41. 10.1111/bcp.12616 25727860 PMC4541971

[218] Testani JM Khera AV St John Sutton MG Keane MG Wiegers SE Shannon RP Effect of right ventricular function and venous congestion on cardiorenal interactions during the treatment of decompensated heart failure. *Am J Cardiol.* (2010) 105:511–6. 10.1016/j.amjcard.2009.10.020 20152246 PMC2995805

[219] Dupont M Mullens W Finucan M Taylor DO Starling RC Tang WHW. Determinants of dynamic changes in serum creatinine in acute decompensated heart failure: the importance of blood pressure reduction during treatment. *Eur J Heart Fail.* (2013) 15:433–40. 10.1093/eurjhf/hfs209 23288912

[220] Aronson D Abassi Z Allon E Burger AJ. Fluid loss, venous congestion, and worsening renal function in acute decompensated heart failure. *Eur J Heart Fail.* (2013) 15:637–43. 10.1093/eurjhf/hft036 23475780

[221] Nohria A Hasselblad V Stebbins A Pauly DF Fonarow GC Shah M Cardiorenal interactions: insights from the ESCAPE trial. *J Am Coll Cardiol.* (2008) 51:1268–74. 10.1016/j.jacc.2007.08.072 18371557

[222] Gelman S. Venous function and central venous pressure: a physiologic story. *Anesthesiology.* (2008) 108:735–48. 10.1097/ALN.0b013e3181672607 18362606

[223] Zymliński R Dierckx R Biegus J Vanderheyden M Bartunek J Ponikowski P. Novel IVC doraya catheter provides congestion relief in patients with acute heart failure. *JACC Basic Transl Sci.* (2022) 7:326–7. 10.1016/j.jacbts.2022.02.013 35411326 PMC8993904

[224] Mohmand H Goldfarb S. Renal dysfunction associated with intra-abdominal hypertension and the abdominal compartment syndrome. *J Am Soc Nephrol.* (2011) 22:615–21. 10.1681/ASN.2010121222 21310818

[225] De Waele JJ De Laet I Kirkpatrick AW Hoste E. Intra-abdominal hypertension and abdominal compartment syndrome. *Am J kidney Dis Off J Natl Kidney Found.* (2011) 57:159–69. 10.1053/j.ajkd.2010.08.034 21184922

[226] Abu-Saleh N Aronson D Khamaisi M Khoury EE Awad H Kabala A Increased Intra-abdominal pressure induces acute kidney injury in an experimental model of congestive heart failure. *J Card Fail.* (2019) 25:468–78. 10.1016/j.cardfail.2019.03.008 30880249

[227] Mullens W Abrahams Z Francis GS Taylor DO Starling RC Tang WHW. Prompt reduction in intra-abdominal pressure following large-volume mechanical fluid removal improves renal insufficiency in refractory decompensated heart failure. *J Card Fail.* (2008) 14:508–14. 10.1016/j.cardfail.2008.02.010 18672199

[228] Verbrugge FH Dupont M Steels P Grieten L Malbrain M Tang WHW Abdominal contributions to cardiorenal dysfunction in congestive heart failure. *J Am Coll Cardiol.* (2013) 62:485–95. 10.1016/j.jacc.2013.04.070 23747781

[229] Mullens W Abrahams Z Skouri HN Francis GS Taylor DO Starling RC Elevated intra-abdominal pressure in acute decompensated heart failure: a potential contributor to worsening renal function? *J Am Coll Cardiol.* (2008) 51:300–6. 10.1016/j.jacc.2007.09.043 18206740

[230] Bradley SE Bradley GP. The effect of increased intra-abdominal pressure on renal function in man. *J Clin Invest.* (1947) 26:1010–22. 10.1172/JCI101867 16695476 PMC439406

[231] Bloomfield GL Blocher CR Fakhry IF Sica DA Sugerman HJ. Elevated intra-abdominal pressure increases plasma renin activity and aldosterone levels. *J Trauma.* (1997) 42:995–7. 10.1097/00005373-199706000-00002 9210531

[232] Patel DM Connor MJJ. Intra-abdominal hypertension and abdominal compartment syndrome: an underappreciated cause of acute kidney injury. *Adv Chronic Kidney Dis.* (2016) 23:160–6. 10.1053/j.ackd.2016.03.002 27113692

[233] Zile MR Bennett TD St John Sutton M Cho YK Adamson PB Aaron MF Transition from chronic compensated to acute decompensated heart failure: pathophysiological insights obtained from continuous monitoring of intracardiac pressures. *Circulation.* (2008) 118:1433–41. 10.1161/CIRCULATIONAHA.108.783910 18794390

[234] Koell B Zotter-Tufaro C Duca F Kammerlander AA Aschauer S Dalos D Fluid status and outcome in patients with heart failure and preserved ejection fraction. *Int J Cardiol.* (2017) 230:476–81. 10.1016/j.ijcard.2016.12.080 28062131 PMC6197425

[235] Van Aelst LNL Arrigo M Placido R Akiyama E Girerd N Zannad F Acutely decompensated heart failure with preserved and reduced ejection fraction present with comparable haemodynamic congestion. *Eur J Heart Fail.* (2018) 20:738–47. 10.1002/ejhf.1050 29251818

[236] Cotter G Metra M Milo-Cotter O Dittrich HC Gheorghiade M. Fluid overload in acute heart failure–re-distribution and other mechanisms beyond fluid accumulation. *Eur J Heart Fail.* (2008) 10:165–9. 10.1016/j.ejheart.2008.01.007 18279771

[237] Aronson D. The interstitial compartment as a therapeutic target in heart failure. *Front Cardiovasc Med.* (2022) 9:933384. 10.3389/fcvm.2022.933384 36061549 PMC9428749

[238] Fallick C Sobotka PA Dunlap ME. Sympathetically mediated changes in capacitance: redistribution of the venous reservoir as a cause of decompensation. *Circ Heart Fail.* (2011) 4:669–75. 10.1161/CIRCHEARTFAILURE.111.961789 21934091

[239] Cotter G Felker GM Adams KF Milo-Cotter O O’Connor CM. The pathophysiology of acute heart failure–is it all about fluid accumulation? *Am Heart J.* (2008) 155:9–18. 10.1016/j.ahj.2006.02.038 18082483

[240] Rosch S Kresoja K-P Besler C Fengler K Schöber AR von Roeder M Characteristics of heart failure with preserved ejection fraction across the range of left ventricular ejection fraction. *Circulation.* (2022) 146:506–18. 10.1161/CIRCULATIONAHA.122.059280 35862208

[241] Mullens W Damman K Harjola V-P Mebazaa A Brunner-La Rocca H-P Martens P The use of diuretics in heart failure with congestion – A position statement from the Heart Failure Association of the European Society of Cardiology. *Eur J Heart Fail.* (2019) 21:137–55. 10.1002/ejhf.1369 30600580

[242] Hollenberg SM Warner Stevenson L Ahmad T Amin VJ Bozkurt B Butler J 2019 ACC expert consensus decision pathway on risk assessment, management, and clinical trajectory of patients hospitalized with heart failure: a report of the American College of Cardiology solution set oversight committee. *J Am Coll Cardiol.* (2019) 74:1966–2011. 10.1016/j.jacc.2019.08.001 31526538

[243] Rodríguez-Espinosa D Guzman-Bofarull J De La Fuente-Mancera JC Maduell F Broseta JJ Farrero M. Multimodal strategies for the diagnosis and management of refractory congestion. an integrated cardiorenal approach. *Front Physiol.* (2022) 13:913580. 10.3389/fphys.2022.913580 35874534 PMC9304751

[244] Bart BA Goldsmith SR Lee KL Redfield MM Felker GM O’Connor CM Cardiorenal rescue study in acute decompensated heart failure: rationale and design of CARRESS-HF, for the Heart Failure Clinical Research Network. *J Card Fail.* (2012) 18:176–82. 10.1016/j.cardfail.2011.12.009 22385937 PMC3503538

[245] Aronson D Burger AJ. Diuretic response: clinical and hemodynamic predictors and relation to clinical outcome. *J Card Fail.* (2016) 22:193–200. 10.1016/j.cardfail.2015.07.006 26209003

[246] Ter Maaten JM Rao VS Hanberg JS Perry Wilson F Bellumkonda L Assefa M Renal tubular resistance is the primary driver for loop diuretic resistance in acute heart failure. *Eur J Heart Fail.* (2017) 19:1014–22. 10.1002/ejhf.757 28105769 PMC6231236

[247] Paterna S Di Gaudio F La Rocca V Balistreri F Greco M Torres D Hypertonic saline in conjunction with high-dose furosemide improves dose-response curves in worsening refractory congestive heart failure. *Adv Ther.* (2015) 32:971–82. 10.1007/s12325-015-0254-9 26521190 PMC4635178

[248] Cox ZL Hung R Lenihan DJ Testani JM. Diuretic strategies for loop diuretic resistance in acute heart failure: the 3T trial. *JACC Heart Fail.* (2020) 8:157–68. 10.1016/j.jchf.2019.09.012 31838029 PMC7058489

[249] Jentzer JC DeWald TA Hernandez AF. Combination of loop diuretics with thiazide-type diuretics in heart failure. *J Am Coll Cardiol.* (2010) 56:1527–34. 10.1016/j.jacc.2010.06.034 21029871

[250] Mullens W Dauw J Martens P Verbrugge FH Nijst P Meekers E Acetazolamide in acute decompensated heart failure with volume overload. *N Engl J Med.* (2022). 10.1056/NEJMoa2203094 [Epub ahead of print]. 36027559

[251] Tamaki S Yamada T Watanabe T Morita T Furukawa Y Kawasaki M Effect of empagliflozin as an add-on therapy on decongestion and renal function in patients with diabetes hospitalized for acute decompensated heart failure: a prospective randomized controlled study. *Circ Heart Fail.* (2021) 14:e007048. 10.1161/CIRCHEARTFAILURE.120.007048 33663235

[252] Griffin M Rao VS Ivey-Miranda J Fleming J Mahoney D Maulion C Empagliflozin in heart failure: diuretic and cardiorenal effects. *Circulation.* (2020) 142:1028–39. 10.1161/CIRCULATIONAHA.120.045691 32410463 PMC7521417

[253] Schulze PC Bogoviku J Westphal J Aftanski P Haertel F Grund S Effects of early empagliflozin initiation on diuresis and kidney function in patients with acute decompensated heart failure (EMPAG-HF). *Circulation.* (2022) 146:289–98. 10.1161/CIRCULATIONAHA.122.059038 35766022

[254] Mullens W Verbrugge FH Nijst P Martens P Tartaglia K Theunissen E Rationale and design of the ADVOR (acetazolamide in decompensated heart failure with volume overload) trial. *Eur J Heart Fail.* (2018) 20:1591–600. 10.1002/ejhf.1307 30238574

[255] Goldsmith SR Burkhoff D Gustafsson F Voors A Zannad F Kolkhof P Dual vasopressin receptor antagonism to improve congestion in patients with acute heart failure: design of the AVANTI trial. *J Card Fail.* (2021) 27:233–41. 10.1016/j.cardfail.2020.10.007 33188886

[256] Greene SJ Velazquez EJ Anstrom KJ Eisenstein EL Sapp S Morgan S Pragmatic design of randomized clinical trials for heart failure: rationale and design of the TRANSFORM-HF trial. *JACC Heart Fail.* (2021) 9:325–35. 10.1016/j.jchf.2021.01.013 33714745 PMC8087639

[257] Cox ZL Collins SP Aaron M Hernandez GA Iii ATM Davidson BT Efficacy and safety of dapagliflozin in acute heart failure: rationale and design of the DICTATE-AHF trial. *Am Heart J.* (2021) 232:116–24. 10.1016/j.ahj.2020.10.071 33144086

[258] Biegus J Zymlinski R Siwolowski P Testani J Szachniewicz J Tycińska A Controlled decongestion by reprieve therapy in acute heart failure: results of the TARGET-1 and TARGET-2 studies. *Eur J Heart Fail.* (2019) 21:1079–87. 10.1002/ejhf.1533 31127666

[259] Rodés-Cabau J Bernier M Amat-Santos IJ Ben Gal T Nombela-Franco L García Del Blanco B Interatrial shunting for heart failure: early and late results from the first-in-human experience with the V-wave system. *JACC Cardiovasc Interv.* (2018) 11:2300–10. 10.1016/j.jcin.2018.07.001 30391390

[260] Ponikowski P Voors AA Anker SD Bueno H Cleland JGF Coats AJS 2016 ESC guidelines for the diagnosis and treatment of acute and chronic heart failure: the task force for the diagnosis and treatment of acute and chronic heart failure of the European Society of Cardiology (ESC)developed with the special contribution o. *Eur Heart J.* (2016) 37:2129–200. 10.1093/eurheartj/ehw128 27206819

[261] Clark H Krum H Hopper I. Worsening renal function during renin-angiotensin-aldosterone system inhibitor initiation and long-term outcomes in patients with left ventricular systolic dysfunction. *Eur J Heart Fail.* (2014) 16:41–8. 10.1002/ejhf.13 24453097

[262] Epstein M. Aldosterone and mineralocorticoid receptor signaling as determinants of cardiovascular and renal injury: from Hans Selye to the present. *Am J Nephrol.* (2021) 52:209–16. 10.1159/000515622 33857953

[263] Rocha R Stier CTJ. Pathophysiological effects of aldosterone in cardiovascular tissues. *Trends Endocrinol Metab.* (2001) 12:308–14. 10.1016/s1043-2760(01)00432-5 11504670

[264] Pitt B Zannad F Remme WJ Cody R Castaigne A Perez A The effect of spironolactone on morbidity and mortality in patients with severe heart failure. Randomized aldactone evaluation study investigators. *N Engl J Med.* (1999) 341:709–17. 10.1056/NEJM199909023411001 10471456

[265] Epstein M. Renin-angiotensin-aldosterone system inhibition and mineralocorticoid receptor antagonists: the overriding importance of enablers and dampers. *Kidney Int Rep.* (2021) 6:869–71. 10.1016/j.ekir.2021.01.032 33912739 PMC8071656

[266] Testani JM Kimmel SE Dries DL Coca SG. Prognostic importance of early worsening renal function after initiation of angiotensin-converting enzyme inhibitor therapy in patients with cardiac dysfunction. *Circ Heart Fail.* (2011) 4:685–91. 10.1161/CIRCHEARTFAILURE.111.963256 21903907 PMC3248247

[267] McDonagh TA Metra M Adamo M Gardner RS Baumbach A Böhm M 2021 ESC guidelines for the diagnosis and treatment of acute and chronic heart failure. *Eur Heart J.* (2021) 42:3599–726. 10.1093/eurheartj/ehab368 34447992

[268] Tsutsui H. Recent advances in the pharmacological therapy of chronic heart failure: evidence and guidelines. *Pharmacol Ther.* (2022) 238:108185. 10.1016/j.pharmthera.2022.108185 35413307

[269] Braunwald E. Gliflozins in the management of cardiovascular disease. *N Engl J Med.* (2022) 386:2024–34. 10.1056/NEJMra2115011 35613023

[270] Aguilar-Gallardo JS Correa A Contreras JP. Cardio-renal benefits of sodium-glucose co-transporter 2 inhibitors in heart failure with reduced ejection fraction: mechanisms and clinical evidence. *Eur Heart J Cardiovasc Pharmacother.* (2022) 8:311–21. 10.1093/ehjcvp/pvab056 34264341

[271] McMurray JJV Solomon SD Inzucchi SE Køber L Kosiborod MN Martinez FA Dapagliflozin in patients with heart failure and reduced ejection fraction. *N Engl J Med.* (2019) 381:1995–2008. 10.1056/NEJMoa1911303 31535829

[272] Packer M Anker SD Butler J Filippatos G Pocock SJ Carson P Cardiovascular and renal outcomes with empagliflozin in heart failure. *N Engl J Med.* (2020) 383:1413–24. 10.1056/NEJMoa2022190 32865377

[273] Petrie MC Verma S Docherty KF Inzucchi SE Anand I Belohlávek J Effect of dapagliflozin on worsening heart failure and cardiovascular death in patients with heart failure with and without diabetes. *JAMA.* (2020) 323:1353–68. 10.1001/jama.2020.1906 32219386 PMC7157181

[274] DeFronzo RA Norton L Abdul-Ghani M. Renal, metabolic and cardiovascular considerations of SGLT2 inhibition. *Nat Rev Nephrol.* (2017) 13:11–26. 10.1038/nrneph.2016.170 27941935

[275] Granger CB McMurray JJV Yusuf S Held P Michelson EL Olofsson B Effects of candesartan in patients with chronic heart failure and reduced left-ventricular systolic function intolerant to angiotensin-converting-enzyme inhibitors: the CHARM-Alternative trial. *Lancet.* (2003) 362:772–6. 10.1016/S0140-6736(03)14284-5 13678870

[276] McMurray JJV Packer M Desai AS Gong J Lefkowitz MP Rizkala AR Dual angiotensin receptor and neprilysin inhibition as an alternative to angiotensin-converting enzyme inhibition in patients with chronic systolic heart failure: rationale for and design of the prospective comparison of ARNI with ACEI to determine impac. *Eur J Heart Fail.* (2013) 15:1062–73. 10.1093/eurjhf/hft052 23563576 PMC3746839

[277] Buggey J Mentz RJ DeVore AD Velazquez EJ. Angiotensin receptor neprilysin inhibition in heart failure: mechanistic action and clinical impact. *J Card Fail.* (2015) 21:741–50. 10.1016/j.cardfail.2015.07.008 26209000

[278] Sica DA. Sodium and water retention in heart failure and diuretic therapy: basic mechanisms. *Cleve Clin J Med.* (2006) 73(Suppl. 2):S2–7; discussion S30–3. 10.3949/ccjm.73.suppl_2.s2 16786906

